# Bio-inspired multispectral camouflage material for microwave, infrared, and visible bands based on single hierarchical metasurface

**DOI:** 10.1515/nanoph-2025-0024

**Published:** 2025-05-01

**Authors:** Shiju Liu, Congyang Zhou, Ruiyang Tan, Mengqi Han, Zhijing Wu, Ping Chen

**Affiliations:** 12581 Nanjing University, Nanjing, China; Suzhou Laboratory, Suzhou, China

**Keywords:** multispectral camouflage, microwave, infrared, metasurface, metamaterial

## Abstract

Nature can significantly inspire humans. Chameleons, jellyfish, and many other creatures use unique camouflage methods. Multispectral camouflage materials are highly desirable to against progressive multispectral detection. The proposed structure should be simple and highly transparent to ensure a wide application range. In this study, a bio-inspired multispectral camouflage material with visible transparency, microwave diffusion, and infrared (IR) camouflage was designed, fabricated, and tested. Multispectral camouflage performance was achieved on a single metasurface by the following steps: First, a nanoscale multilayered film consisting of an oxide and metal was unitized to achieve a low IR emissivity and high visible transmittance. Then, two units were designed to obtain a phase difference, thus realizing the microwave diffusion performance. Based on the relationship between the area filling fraction and IR emissivity, the units can perform puzzled imaging under an IR thermal camera. The structural parameters were calculated and optimized through an equivalent circuit model-based artificial intelligence algorithm. Then, a 10 dB reduction in radar cross section from 7 GHz to 16 GHz, a puzzled IR thermal image, and a high optical transmittance (>0.7) were achieved. The work provides significant guidance for the design and fabrication of multispectral camouflage materials.

## 1 Introduction

Camouflage is a natural behavior that protects living organisms [1]. The microstructure of surface tissues formed by biological evolution and natural selection is an important platform for camouflaging organisms [2], [3]. For example, chameleons control the stretching or relaxation of nanoscale crystals on their body surfaces to create colorful and disruptive patterns that blend with their surrounding background [4]. The body of a jellyfish has evolved a special three-layer structure in which the middle layer is the lamella layer. This layer is mainly composed of water, which makes the body more transparent such that it can better integrate with the marine environment [5]. These microstructure-based disguises of living organisms inspired humans to develop artificial camouflage materials and devices including camouflage clothing [6], transparent wood [7], and invisibility cloaking [8], [9]. Nonetheless, the processes of formation and natural evolution of the microstructure of surface tissues of living organisms are exceptionally long and generally require millions of years [4], [10]. Moreover, the camouflage strategies of organisms tend to target only one spectrum, typically visible light. In recent years, rapidly emerging technologies such as metamaterials [11], [12], [13], [14], [15], [16], [17], [18], [19], [20], [21], [22], [23], [24], micro–nano processing [25], [26], and artificial intelligence (AI) [27], [28], [29], [30] have altered the paradigm of how humans design and manufacture materials [25], [31]. Metamaterials constitute an emerging new type of artificial material based on designed subwavelength elements. These enable frequency-selective electromagnetic (EM) responses in different bands [11], [12], [13], [14], [15], [16], [17], [18], [19], [20], [21], [22], [23], [24]. Through AI-based design and micro–nano fabrication of artificial structures, humans can eliminate the constraints of natural evolution in time and space and rapidly design and implement camouflage materials from the microwave to visible light bands [32], [33], [34]. In particular, through multiscale microstructure design, camouflage functions of different bands can be realized simultaneously in a single metamaterial. This capability is effective for the rapidly evolving advanced multiband detection technology, both military and civilian.

Over the past decade, significant progress has been achieved in the utilization of metamaterials to realize multispectral camouflaged materials. For example, Zhu et al. introduced a multispectral camouflage metamaterial with a cascade structure consisting of a ZnS/Ge multilayer for wavelength-selective infrared (IR) emission and a lossy metasurface for microwave absorption [35]. Liu et al. demonstrated a multilayered metamaterial with a ferromagnetic material to achieve broadband microwave camouflage and a low IR emissivity. The entire structure consists of an IR shield layer, a transition layer, a microwave absorber including a resistive microwave absorption layer, a dielectric spacer, a ferromagnetic material, and a metallic backboard from the top to the bottom [36]. Feng et al. proposed a multilayered microwave absorber enclosed by a micro/nanofabricated surface to achieve multispectral camouflage. The surface has a low IR emissivity, high laser scattering, and high microwave transmission [37]. It is worth mentioning that the fundamental configuration deployed in all the above studies is a cascaded structure of metamaterials working in the microwave and IR bands. This configuration is selected because microwave-absorbing materials usually have high IR emissivity, and low IR emissivity materials such as metals have high reflectivity to microwaves. To resolve this conflict, researchers have proposed a cascaded structure in which an IR camouflage metasurface with a low IR emissivity and high microwave transmittance is placed on top of the microwave-absorbing metamaterial. However, cascaded structures increase the complexity of design and fabrication, thereby incurring higher costs. Li et al. recently proposed a single-layer metasurface displaying both microwave absorption and low IR emissivity [38]. However, they utilized a conductive film to implement the metasurface unit for a low IR emissivity. This also yielded a microwave absorber with a narrow absorption bandwidth of 0.4 GHz. Therefore, multispectral metamaterials with single-layer structures and broad microwave camouflage bands are still worth evaluating because bandwidth is crucial for microwave camouflage.

In most previous studies, the IR stealth strategy for metamaterials involved the deployment of metasurfaces with low IR emissivity. Such a strategy is effective for the IR camouflage of targets whose temperature is higher than the ambient one. This is because a low emissivity implies a low IR radiation signal according to the Stefan–Boltzmann law [33]. The main approach to achieving a low IR emissivity is to utilize materials with high reflectivity of EM waves in the IR band to construct IR metasurfaces, such as metals, conductive oxides, and two-dimensional materials [39]. However, this camouflage strategy may fail in certain complex scenarios and may even cause the IR characteristics of the target to become more exposed to the thermal imager [39]. For example, a target with a low IR emissivity in a city may reflect IR waves that radiate from the ground and form a hot spot on a thermal imager when its temperature is lower than that of the ground. Therefore, it is worthwhile to evaluate the construction of a metasurface that can achieve IR camouflage. Recently, researchers designed a digital IR emissivity disguise surface as the top layer of a multispectral camouflage material [40], [41].

In this study, we proposed and demonstrated a single metasurface with broadband microwave camouflage and IR camouflage. It is based on a visible transparent multilayered oxide metal film and a cross-scale design. ZnO-doped Al (AZO) and Ag were selected as the common and widely used materials. First, we selected diffusion as the microwave camouflage strategy after analyzing the relationship between the camouflage principle and the number of metasurface layers. We calculated and analyzed the configuration of the multilayered film to determine the best configuration for an IR camouflage material (IRCM) with a high visible transmittance. With regard to the neuroma parameters and goals, we adopted a genetic algorithm (GA) to optimize the structural parameters simultaneously. Finally, the IRCM was fabricated using magnetron sputtering technology. After direct laser writing on one side of the IRCM, the multispectral camouflage metasurface (MSCM) performed well. It is worth mentioning that multispectral camouflage performance was achieved on a single metasurface. Moreover, the MSCM was analyzed in the framework of an equivalent circuit model (ECM) covering the visible, IR, and microwave bands. The structural parameters at the nanometer, micrometer, and millimeter scales were optimized using an ECM-based GA. The prototype sample was then tested. The results show that the MSCM has wide applications in multispectral camouflage.

## 2 Result, mechanism, and modeling

**Figure 1:**
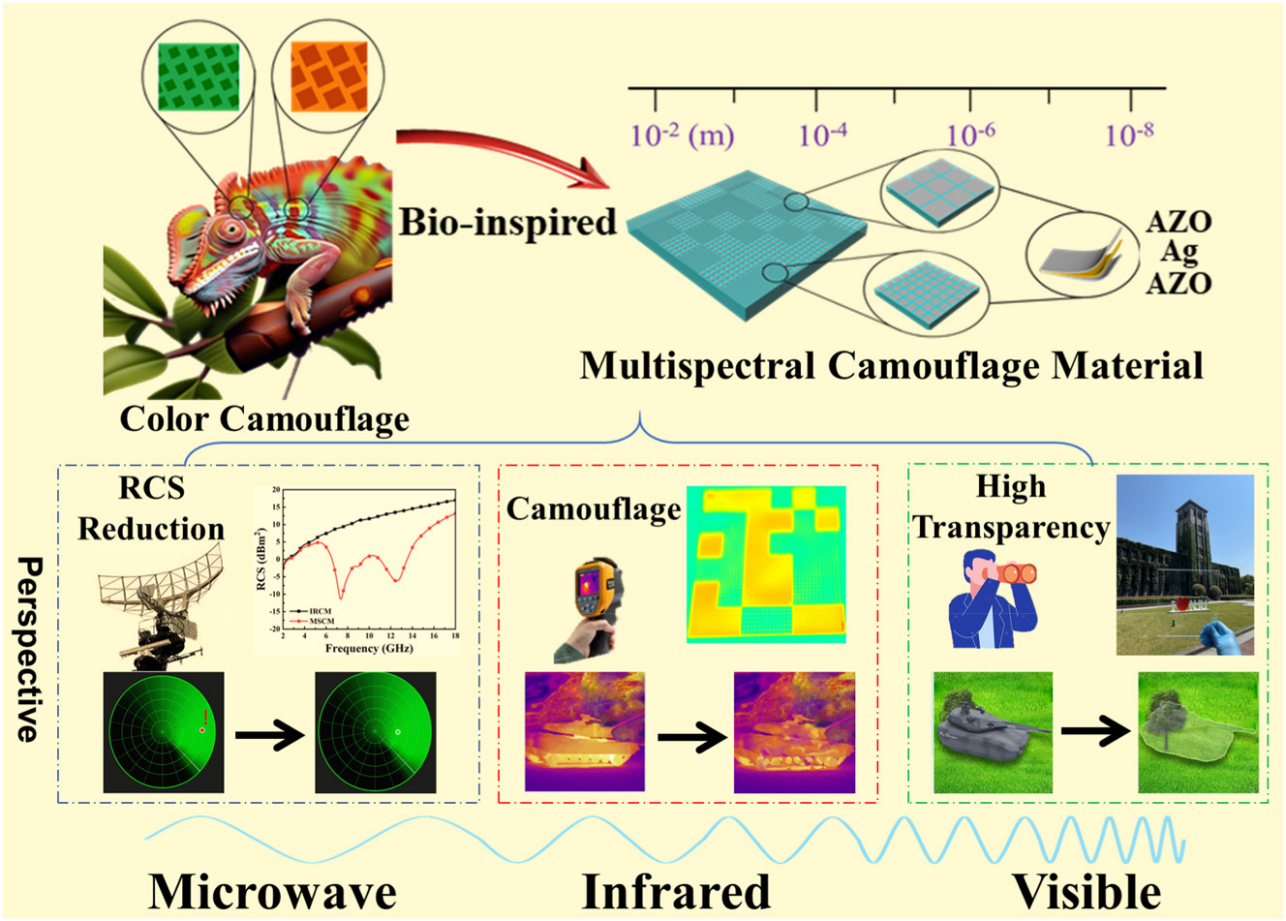
Schematic of the proposed multispectral camouflage material, including its total structure and unit cells. Broadband microwave diffusion, IR camouflage, and high visible transmittance are achieved in a single metasurface. The proposed MSCM would have many applications in multispectral camouflage.

Figure 1 shows a schematic diagram of the proposed MSCM. It is a metasurface containing two atoms with different IR emissivity and microwave reflection phases. MSCMs can achieve IR camouflage and microwave scattering. Owing to the use of oxide–metal–oxide films, MSCMs display high visible-light transmittance. These have several applications in multispectral camouflage. The detailed design and analysis processes are described below.

### 2.1 Single metasurface for microwave and IR camouflage

Diffuse-scattering metasurfaces can reduce the radar cross section (RCS) in the backward or specular direction by randomly scattering the EM energy in various directions in space [13]. This is an alternative microwave camouflage method for absorption. This mechanism relies only on the phase cancellation between different elements rather than the absorption of incident EM waves. This enables the use of good conductors in metasurface elements. Good conductors also have a low emissivity in the IR band, which allows for IR camouflage. Therefore, we propose a strategy to achieve camouflage in both IR and microwave bands on a single metasurface by designing cross-scale structures. The mechanism of RCS reduction (RCSR) achieved by a diffused scattering metasurface can be described using the antenna array theory. For a ground plane, the array factor (AF) can be represented by Eq. (1) [42]:

(1) $$\text{AF}={\text{I}}_{0}\overset{M}{\underset{m=1}{\sum }}\overset{N}{\underset{n=1}{\sum }}e^{j\left[\left(m-1\right)\left(kd_{x}\sin\theta \cos\emptyset +\beta_{x}\right)+\left(n-1\right)\left(kd_{y}\sin\theta \sin\emptyset +\beta_{y}\right)\right]}$$

where *β*_*x*_ and *β*_*y*_ are the phase shifts between the elements in the x- and y-directions, respectively. *d*_*x*_ and *d*_*y*_ are the spacings between the elements along the x- and y-axes, respectively. M and N are the numbers of elements in the x- and y-directions, respectively. The total RCSR, compared with that of a perfect electrical conductor (PEC), has the following formation:

(2) $$\text{RCSR}=10\mathrm{lg}\left|\frac{\underset{r\to \infty }{\lim}\left(\frac{4\pi r^{2}{\left|E^{s}\right|}^{2}}{{\left|E^{i}\right|}^{2}}\right)}{\underset{r\to \infty }{\lim}\left(4\pi r^{2}\right)}\right|=10\mathrm{lg}\left(\frac{{\left|E^{s}\right|}^{2}}{{\left|E^{i}\right|}^{2}}\right)dB,$$

where E^s^ and E^i^ are the incident and diffused electric fields of the EM waves, respectively. Specifically, if the diffusion metasurface is composed of only two types of elements, the RCSR in the specular direction can be expressed as follows [19]:

(3) $$\text{RCSR}=20\mathrm{lg}\left(p_{1}r_{1}e^{j\varphi_{1}}+p_{2}r_{2}e^{j\varphi_{2}}\right)dB,$$

where *p*_*1*_, *p*_*2*_ are the filling ratios. *r*_*1*_, *r*_*2*_ and *φ*_*1*_, *φ*_*2*_ are the amplitudes and phases for the reflectivity of each element, respectively. According to Eq. (3), if the phase difference between *φ*_*1*_ and *φ*_*2*_ among 180° ± 37° over a broad frequency range, the metasurface can achieve a broadband −10 dB RCSR in the backward direction even for lossless elements, *r*_1_ = *r*_2_ = 1. The reflection phase of a specific metasurface element can be adjusted by modifying its structure [29]. Therefore, broadband RCSR can be achieved through phase cancellation by designing different unit structures in a metasurface composed of good conductors. Moreover, Eq. (1) indicates that the diffused scattering of EM waves can be realized in backward space by randomly arranging cells with different reflection phases, thus achieving microwave camouflage [43].

With regard to the IR camouflage performance, according to the efficient medium theory, the IR emissivity (E) is related to the area-filling fractions X_1_, X_2_ and IR emissivity E_1_, E_2_ of different materials, as follows [29]:

(4) $$E=E_{1}X_{1}+E_{2}X_{2}.$$

When the composition of a metasurface is determined, altering the filling fractions of the different components can yield different IR emissivity values. If these elements with different IR emissivity have a special arrangement, IR camouflage can be achieved rather than only low IR emissivity. Therefore, if we can design metasurface units whose reflection phases depend directly on the filling ratio of the low IR emissivity conductive materials, IR camouflage and broadband microwave camouflage can be achieved on a single metasurface.

Chameleons can display different colors by contracting or stretching patch structures on their skin. These structures modulate EM waves in the visible band. Inspired by chameleons, we used a patch pattern to achieve microwave diffusion. The structural parameters of the patch pattern, including its period and gap, affect its EM response [29]. Specifically, the capacitance effect between each patch varies with the structural parameters. Therefore, we used a patch pattern to achieve microwave diffusion.

**Figure 2:**
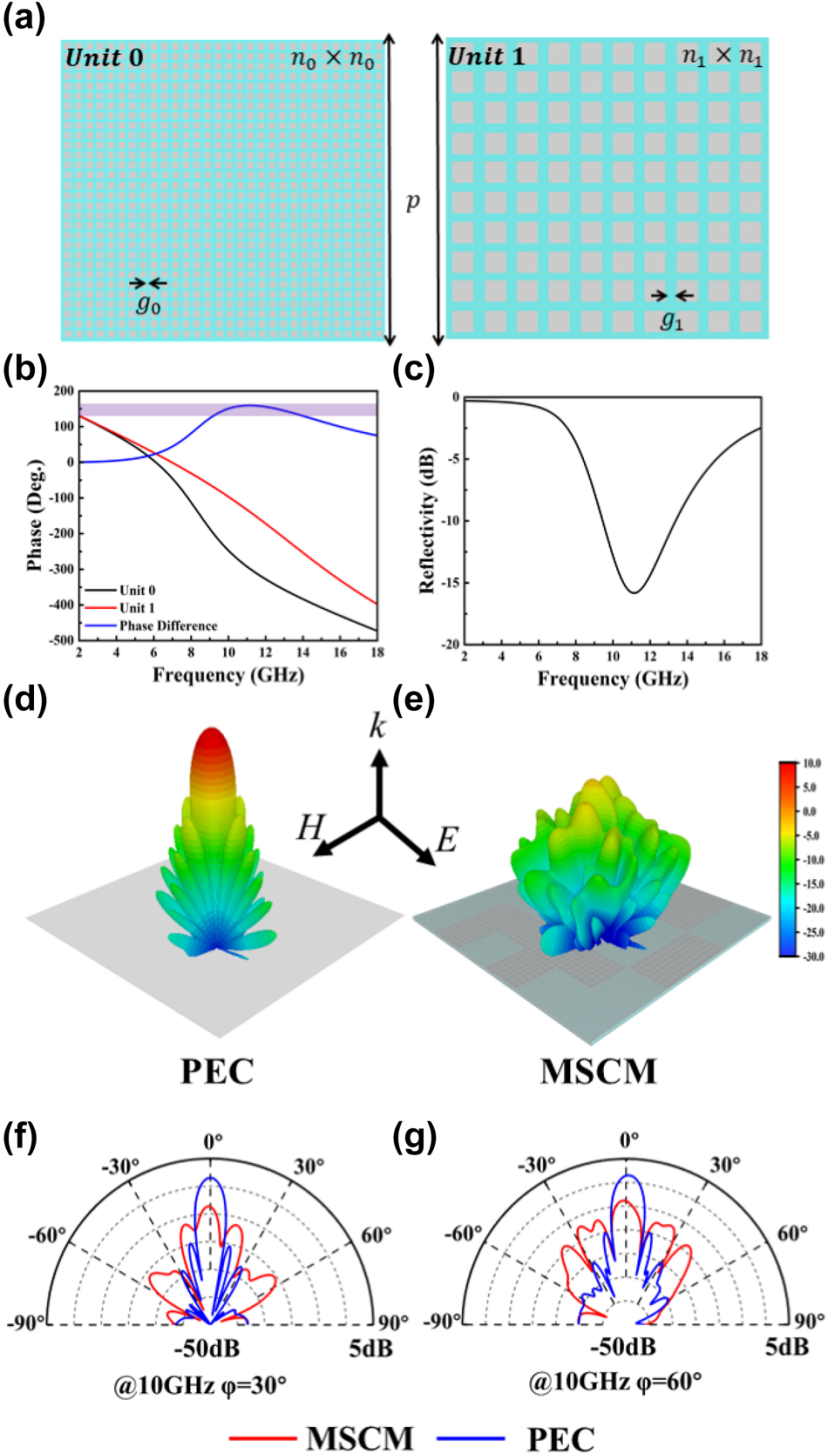
Related information about the proposed MSCM in microwave band (a) top view of units 0 and 1, where p = 30 mm, n0 = 40, g0 = 0.15 mm, n1 = 10, g1 = 0.15 mm, (b) simulated reflection phase by CST of units 0 and 1 and a phase difference of 180°± 37°, (c) simulated reflectivity of the metasurface. Simulated 3D far-field scattering pattern of (d) PEC and (e) the metasurface at 10GHz. Simulated 1D far-field scattering pattern of PEC and the metasurface at 10GHz for the azimuth angles of (f) 30° and (g) 60°.

Figure 2a shows two units: the n_0_ × *n*_0_ and n_1_ × *n*_1_ patches. p is the period of each unit, and g is the gap between each patch. The simulated reflection phases of different unit cells are shown in Figure 2b. A phase difference of 180° ± 37° existed from 9 GHz to 13.5 GHz. Therefore, the reflectivity of the metasurface composed of these two types of units can be calculated according to Eq. (3), which is shown in Figure 2c. The results indicate that the corresponding reflectivity is below −10 dB from 9 GHz to 13.5 GHz. Then, we randomly arranged the different reflective phase unit cells with the same quantity into a two-dimensional array to construct the MSCM. Figure 2d and e shows the 3D far-field scattering pattern of the PEC plate and MSCM under normal incidence at 10 GHz. As shown, the MSCM generates more scattered lobes compared to the PEC plate. The backward lobe intensity of MSCM is lower than that of the PEC plate, indicating that the MSCM achieves a backward RCSR. Figure 2f and g further present the 1D far-field scattering patterns of the MSCM and the PEC plate at 10 GHz for different azimuth angles. The results show that the intensity of main lobe, i.e., the backward lobe of the MSCM, is 13.1 dB lower than that of the PEC plate, while the lobe intensities in other directions are slightly higher than those of the PEC plate. This indicates that the MSCM achieves an RCSR of 13.1 dB at 10 GHz, which is consistent with the reflectivity of the MSCM at 10 GHz shown in Figure 2c. Meanwhile, the total energy scattered by the MSCM in the backward hemisphere is not reduced compared to the PEC plate since lossless patches are deployed. However, due to the interference between unit cells with different reflection phases, the MSCM forms more lobes, thereby achieving microwave camouflage.

For a common low IR emissivity material copper and polymethyl methacrylate (PMMA) substrate, these have IR emissivity of 0.05 and 0.95 [44], respectively. Then, Eq. (4) is modified as follows:

(5) $$\text{E}=0.05\times {\left(1-\frac{g}{p/n}\right)}^{2}+0.95\times \left(1-{\left(1-\frac{g}{p/n}\right)}^{2}\right).$$

**Figure 3:**
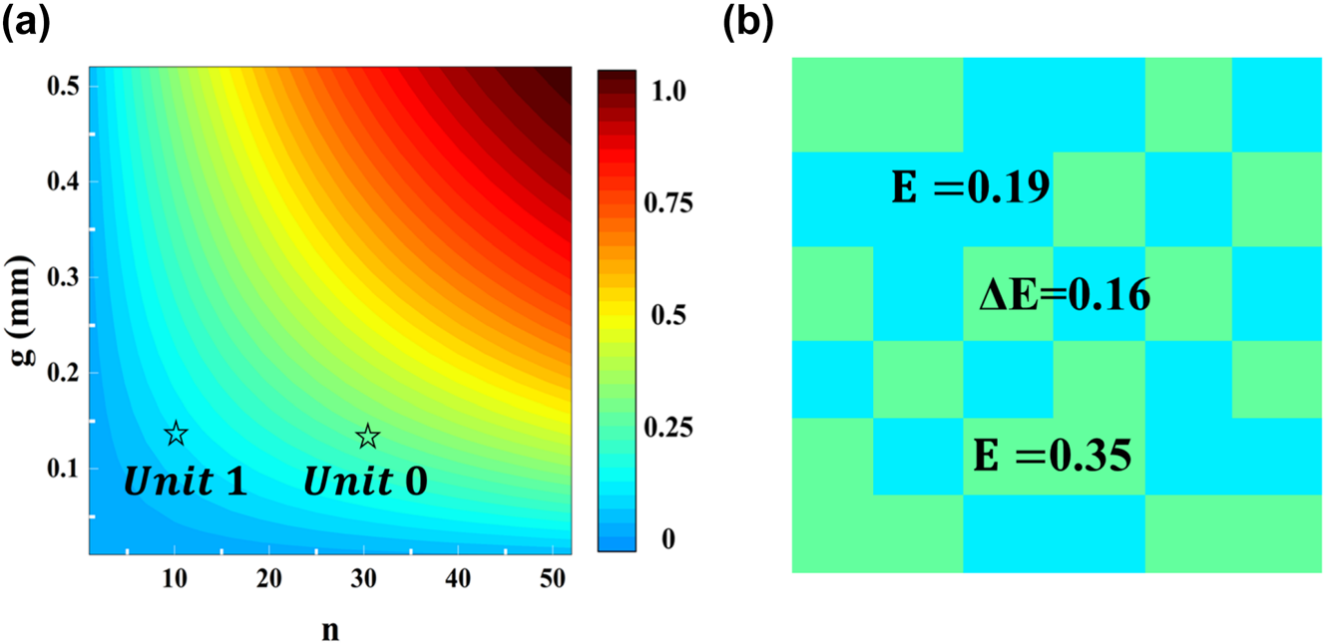
Related information about the proposed MSCM in infrared band (a) relationship between n, g, and E. The black stars represent the IR emissivity corresponding to the structural parameters of two units. (b) Calculated IR thermal imager of the metasurface.

Figure 3a shows the relationship between n, g, and E. Moreover, the color bar represents the image captured by the thermal camera corresponding to the IR emissivity. As marked by the black star in Figure 3a, the IR emissivity of unit cells 0 and 1 are 0.19 and 0.35, respectively. A gap of 0.16 existed between the IR emissivity values. When the ambient temperature is determined, the colors of materials with different emissivity are different under the infrared thermal imager, as shown in Figure 3a. Thus, the proposed metasurface under a thermal imager will display the image shown in Figure 3b, which is a typical digital camouflage pattern.

### 2.2 Design of high transparency conductive film

Optical transparency is a crucial property for various scientific and technological applications including light transmission and optoelectronics [44]. Metals such as gold, silver, and copper are candidate IR camouflage materials. However, these exhibit low visible transmittance, which limits their applications. Currently, multilayer films based on oxide and metal overlays are widely used in visible-light antireflection [45], low IR emission [46], and microwave shielding [47]. These can achieve low IR emissivity and high transparency. As common and widely used materials, Ag and AZO play important roles in many optoelectronic devices owing to their modulating properties on EM waves [48], [49], [50].

For a k layer nonmagnetic multilayer film, the transmission matrix is calculated according to the transmission line theory [51]. The schematic model and ECM model of the oxide and metal overlapping multilayer film are shown in Figure S1 (Supporting Information).

(6) $$\left[\begin{array}{cc} A_{m} & B_{m}\\ C_{m} & D_{m} \end{array}\right]=\overset{k}{\underset{i=1}{\prod }}\left[\begin{array}{cc} \cos\left(\beta_{i}\times h_{i}\right) & j\sin\left(\beta_{i}\times h_{i}\right)/Y_{t_{i}}\\ jY_{t_{i}}\sin\left(\beta_{i}\times h_{i}\right) & \cos\left(\beta_{i}\times h_{i}\right) \end{array}\right],$$

where *h* is the thickness of the layer, *λ* is the wavelength of the working frequency, $\beta =2\pi \sqrt{\varepsilon }/\lambda$ is the corresponding propagation constant, $Y_{t}=\sqrt{\varepsilon }/Z_{0}$ represents the characteristic admittance of the corresponding material, Z_0_ = 377 Ω represents the wave impedance of vacuum, and each layer has its dielectric constant *ɛ* = *ɛ*′ − 1*j* ×*ɛ*″. The optical parameters and corresponding EM parameters of Ag and AZO in IR and visible bands are shown in Figure S2 (a) to (d) (Supporting Information). R and T represent the reflection power and transmission power of the material, respectively. They can be calculated as follows:

(7) $$\text{R}={\left|\frac{A_{m}+B_{m}/Z_{0}-C_{m}Z_{0}-D_{m}}{A_{m}+B_{m}/Z_{0}+C_{m}Z_{0}+D_{m}}\right|}^{2},$$

(8) $$\text{T}={\left|\frac{2}{A_{m}+B_{m}/Z_{0}+C_{m}Z_{0}+D_{m}}\right|}^{2}.$$

According to Kirchhoff’s laws of thermal radiation and energy conservation, the emissivity (E) of a flat material can be expressed as follows [52], [53]:

(9) E=α=1−R−T,

where α represents the absorption power. Usually, multilayered films are fabricated on a PMMA substrate, and the influence of the substrate on the IR emissivity and visible transmittance can be omitted (see Section S1.2 in the Supporting Information for details). Using a typical total oxide thickness of 100 nm and total metal thickness of 10 nm, we calculated the visible transmittance and IR emissivity under different configurations such as oxide (O)–metal (M)–oxide (O) according to Eq. (9). The aim was to initially obtain an ideal configuration.

**Figure 4:**
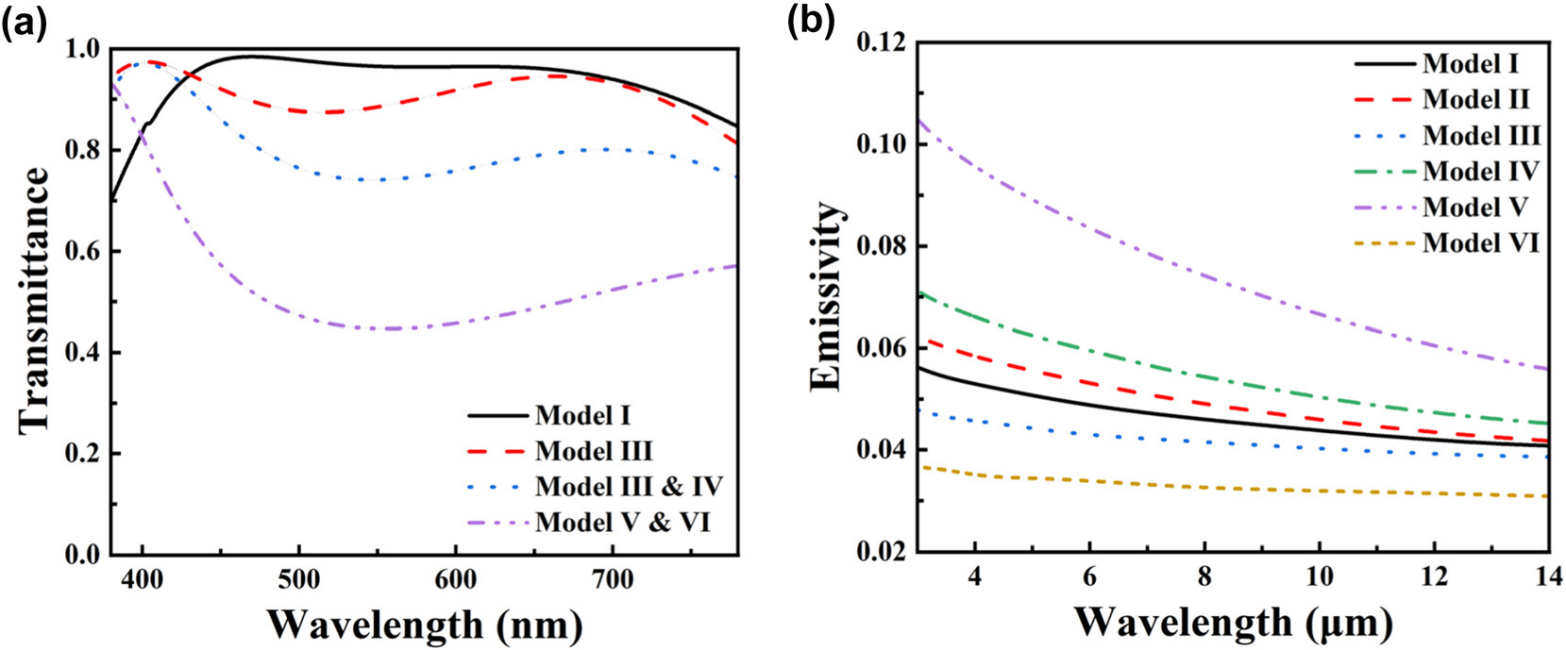
Design of the multilayer films: (a) visible transmittance and (b) IR emissivity of different configurations.

**Figure 5:**
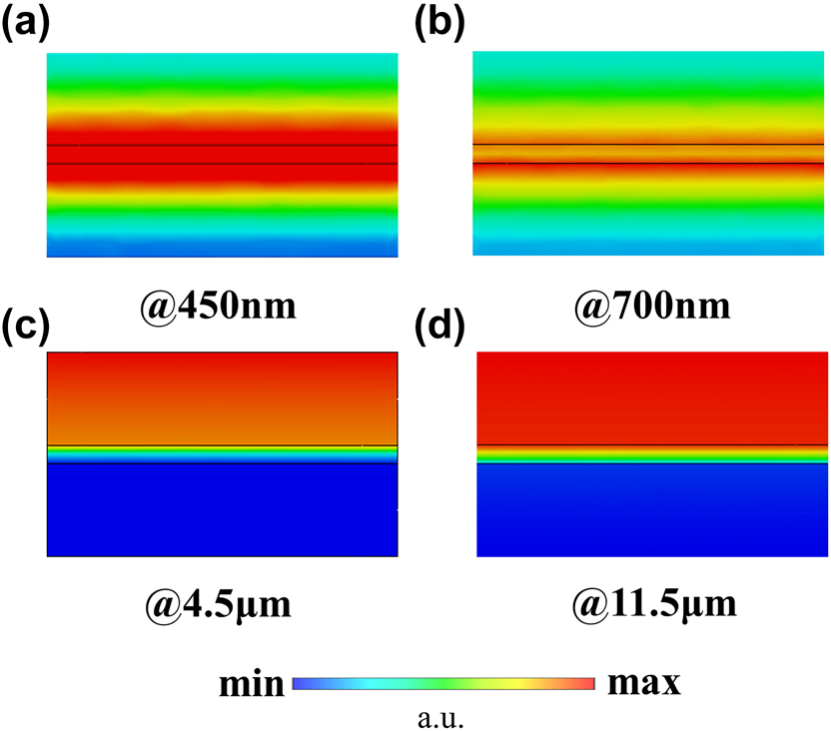
Normalized magnetic field distributions at (a) 450 nm, (b) 700 nm, (c) 4.5 μm, and (d) 11.5 μm of Model I.

As shown in Figure 4a, Model I exhibits the best visible light transmission performance, followed by Model II. Reference [54] explains the mechanism behind this result. This is due to the fact that Ag and AZO have negative and positive dielectric constants, respectively, in the visible light range (see Supplementary Information Figure S2). Therefore, Model I forms a symmetric structure with positive–negative–positive dielectric constants. The EM tunneling effect in such symmetric structure allows the visible light to pass through almost perfectly, thereby achieving higher transmittance. This mechanism is different from the Fabry–Pérot effect since it is independent of the thickness of the negative dielectric constant layer and is also broadband. Model II can be considered as the superposition of two sets of symmetric structures, but the additional interfaces somewhat affect the transmittance, making it suboptimal. The other four models lack this symmetric structure, so their transmittance is inferior to that of Models I and II. Reference [54] also points out that when EM tunneling occurs, the magnetic field localizes near the negative dielectric constant layer. Figure 5a and b shows the normalized magnetic field distribution of the Model I at wavelengths of 450 nm and 700 nm. As shown, the magnetic field indeed localizes near the Ag film.

Figure 4b presents the infrared emissivity performance of each model calculated based on Eq. (6)–(9). In addition, Figure 5c and d displays the normalized magnetic field distribution of Model I at wavelengths of 4.5 μm and 11.5 μm, respectively. It can be observed that the incident electromagnetic waves are completely reflected by the Ag film and do not penetrate into the bottom AZO layer. The magnetic field distribution in the top AZO layer is relatively uniform, indicating a tiny absorption rate of electromagnetic wave energy within it. Therefore, according to Eq. (9), Model I exhibits low infrared emissivity, as shown in Figure 4b. The detailed field distributions of Model II to VI are shown in Section S1.3 (Supporting Information) for further analysis.

Table 1 provides a comprehensive comparison of the performance of these multilayer film models in both the infrared and visible light ranges. Model I and Model III exhibit more balanced advantages. However, Model III is more complex in terms of processing technology compared to Model I, due to its greater number of layers and thinner individual layer thicknesses. Therefore, we selected Model I in this work. The calculated average IR emissivity s and average visible transmittance are 0.048 and 0.86, respectively. The parameter sweeping results are shown in Figure S6 (Supporting Information) to analyze the influence of thickness variation. Furthermore, we calculated the square resistance R_S_ of the proposed film at ambient temperature and pressure, as follows [15]:

(10) $${\text{R}}_{\text{S}}=\frac{1}{\overset{3}{\underset{i=1}{\sum }}\sigma_{i}h_{i}},$$

where σ_i_ and h_i_ are the conductivity and thickness, respectively, of each layer. At ambient temperature and pressure, the conductivities of AZO and Ag are 1 × 10^6^ S/m and 6.3 × 10^7^ S/m [54], respectively. The calculated is approximately 1.1 Ω/sq. Such a low R_S_ means that it exhibits the same EM properties as metals in the microwave range [55]. Therefore, the copper in the original MSCM can be replaced by the AZO–Ag–AZO multilayered film, which has a high visible transparency and low IR emissivity.

**Table 1:** Visible transmittance and IR emissivity of different configurations.

		Model I	Model II	Model III	Model IV	Model V	Model VI
Configuration(nm)		O–M–O	O–M–O–M–O	O–M–O–M	M–O–M–O	O–M	M–O
		50–10–50	25–5–50–5–25	50–5–50–5	5–50–5–50	100–10	10–100
Rank	Visible	#1	#2	#3	#3^a^	#4	#4
	IR	#3	#4	#2	#5	#6	#1

^a^For a reciprocal passive two-port network, the configuration does not affect the transmission coefficient. Therefore, the visible transmittances of Models III and IV and Models V and VI are identical.

### 2.3 Multitarget optimization for multispectral camouflage via genetic algorithm

We have constructed an MSCM with a hierarchical microstructure, featuring a minimum characteristic scale of 10 nm. The target wavelength (band) of such metasurface is from 300 nm (1,000 THz) to 0.15 m (2 GHz), thus extending over six orders of magnitude. This implies that even for full-wave simulation of its microwave performance, a mesh grid at the nm scale is required. Such simulations involve an astronomical computational scale, making it impossible to jointly optimize the multispectral performance of the proposed metasurface in the microwave range using full-wave simulation [31].

As mentioned in Section 2.2, we calculated the responses in the visible and IR bands using ECM. Similarly, we also can establish an ECM of the proposed MSCM [29]. We first calculated the ABCD transmission matrix as follows:

(11) $$\begin{array}{c} \left[\begin{array}{cc} A & B\\ C & D \end{array}\right]=\left[\begin{array}{cc} 1 & 0\\ 1/\left(R_{k}+\frac{1}{j\omega C_{k}}\right) & 1 \end{array}\right]\\ \;\times \left[\begin{array}{cc} \cos\left(\beta \times h\right) & j\sin\left(\beta \times h\right)/Y_{t}\\ jY_{t}\sin\left(\beta \times h\right) & \cos\left(\beta \times h\right) \end{array}\right]\left[\begin{array}{cc} 1 & 0\\ 1/R_{s} & 1 \end{array}\right], \end{array}$$

where ω is the operating angular frequency, $Y_{t}=\sqrt{\varepsilon_{\mathrm{PMMA}}}/Z_{0}$ , and $\beta =2\pi \sqrt{\varepsilon_{\mathrm{PMMA}}}/\lambda$ . k is equal to zero and one and represents each unit cell. The ECM model and calculation process of equivalent parameters *R*_*k*_ and *C*_*k*_ are shown in Section S1.5 (Supporting Information). Then, the reflectivity Γ under normal incident can be calculated as follows:

(12) $$\Gamma =20\mathrm{lg}\left(\left|\frac{A+B/Z_{0}-CZ_{0}-D}{A+B/Z_{0}+CZ_{0}+D}\right|\right)dB.$$

Now that we have the analytical ECMs for the proposed MSCM in the visible, infrared, and microwave bands, we can integrate them with some typical intelligent algorithms to perform inverse design of the metasurface on demand of multispectral performance. The genetic algorithm (GA) has been demonstrated as an efficient algorithm to search for an optimal solution for physical problems. It has been utilized successfully in the design of metamaterials [56], [57], [58], [59], [60], [61]. Therefore, we built a GA-based design program for the MSCM. In this work, the design task consisted of the following five parts:

(1) visible transmittance T > 0.85,
(2) IR emissivity of the film E < 0.05,
(3) difference between the emissivity of units ΔE > 0.25,
(4) microwave reflectivity Γ < −10 dB,
(5) thickness h < 3 mm.

The setting, principle, and progress of the GA program are shown in Section S1.6 (Supporting Information).

**Figure 6:**
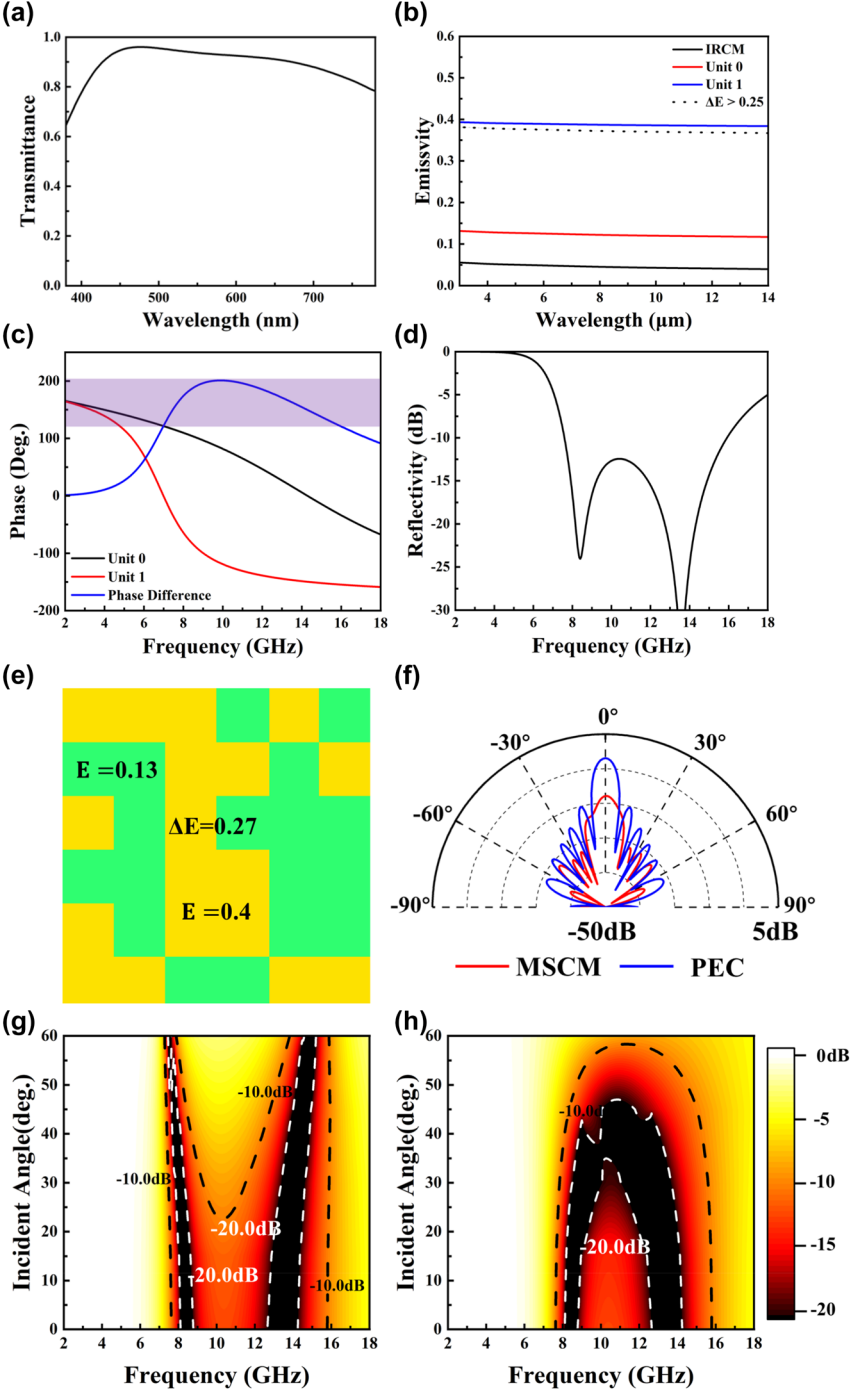
Optimization results including (a) calculated visible transmittance; (b) calculated IR emissivity of the IRCM and each unit; (c) calculated reflection phase of units 0 and 1 (a phase difference of 180° ± 37° is obtained from 7 GHz to 16 GHz); (d) calculated reflectivity of the MSCM; (e) random order and the IR thermal imaginer of the MSCM; (f) simulated 1D far-field scattering pattern of the metasurface at 10 GHz; calculated oblique microwave reflectivity under (g) TE, (h) TM polarization modes.

After completing the optimization, a group of structural parameters extending from millimeters to nanometers was obtained in addition to the multispectral camouflage performance. The optimized result is as follows: h_1_=50 nm, h_2_=12 nm, h_1_=50 nm, h=2.7 mm, g_0_=0.14 mm, g_1_=0.21 mm, n_0_=46, n_1_=7, x=0.5. Figure 6a and b shows the optimized visible transmittance and IR emissivity of the film, with an average transmittance of 0.89 and average emissivity of 0.04. The difference in IR emissivity between units is larger than 0.25. Figure 6c plots the calculated reflection phase of units 0 and 1. The purple region represents the frequency range of microwave reflectivity smaller than −10 dB. The optimized result is shown in Figure 6d, i.e., a −10dB microwave camouflage at 7 GHz–16 GHz. Evidently, the GA program has accomplished the design task. Compared to the original design shown in Figure 2, the optimized version exhibits superior camouflage performance across the visible, infrared, and microwave spectrums.

Moreover, Figure 6e shows the IR thermal imaginer of the MSCM. Herein, the irregular IR camouflage performance would deceive the detectors. Figure 6f plots the 1D far-field scattering pattern of the MSCM at 10 GHz. As shown, the incident EM wave energy is also diffused in different directions. More information about the microwave camouflage mechanism of the optimized MSCM is presented in Section S1.7 (Supporting Information). Moreover, we calculated the microwave reflectivity under oblique incidence according to the method presented in Ref [29]. The calculated results are plotted in Figure 6g and h. As shown, for both transverse electric (TE) and transverse magnetic (TM) polarization modes, the MSCM is still capable of performing microwave camouflage at certain oblique angles of incidence. More details about the far-field scattering patterns under oblique incidence are shown in Section S1.8 (Supporting Information).

## 3 Fabrication, verification, and discussion

**Figure 7:**
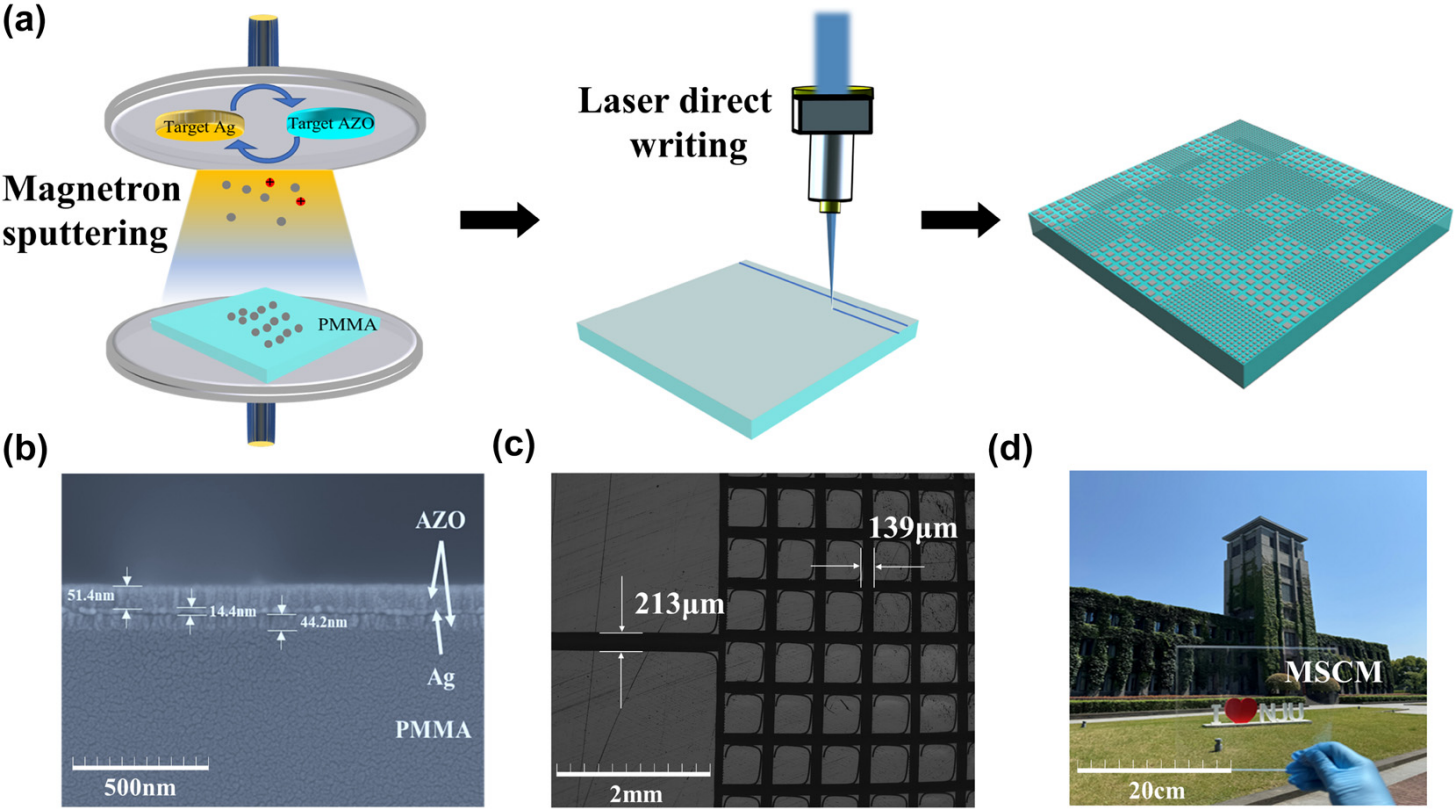
Related information about fabrication process of the MSCM (a) schematic diagrams of fabrication process used for the IRCM and MSCM, including magnetron sputtering and laser direct writing. Photographs of the (b) side view of IRCM at nanoscale, (c) top view of MSCM at microscale, (d) processed sample.

Figure 7a shows the manufacturing procedure of the MSCM: First, a double-polished PMMA was prepared. Then, the AZO/Ag/AZO multilayered film was deposited on the two sides of the PMMA according to the optimized thickness by magnetron sputtering. Finally, one side was processed using laser direct writing technology. The laser line speed is 6,000 mm per minute. The total processing time is about 30 min. Figure 7b shows the side view of the PMMA-based AZO/Ag/AZO multilayered film. As shown, the thicknesses of the layers were 51.4 nm, 14.4 nm, and 44.2 nm, respectively. These are close to the designed values. The surface characterization is shown in Figure 7c. Here, the patch structures are clearly visible, and the gap between each patch is close to the optimized results. Figure 7d shows a visible image of the MSCM. Evidently, it has a high visible transmittance. More information about the fabrication procession is shown in Section S2.1 (Supporting information).

**Figure 8:**
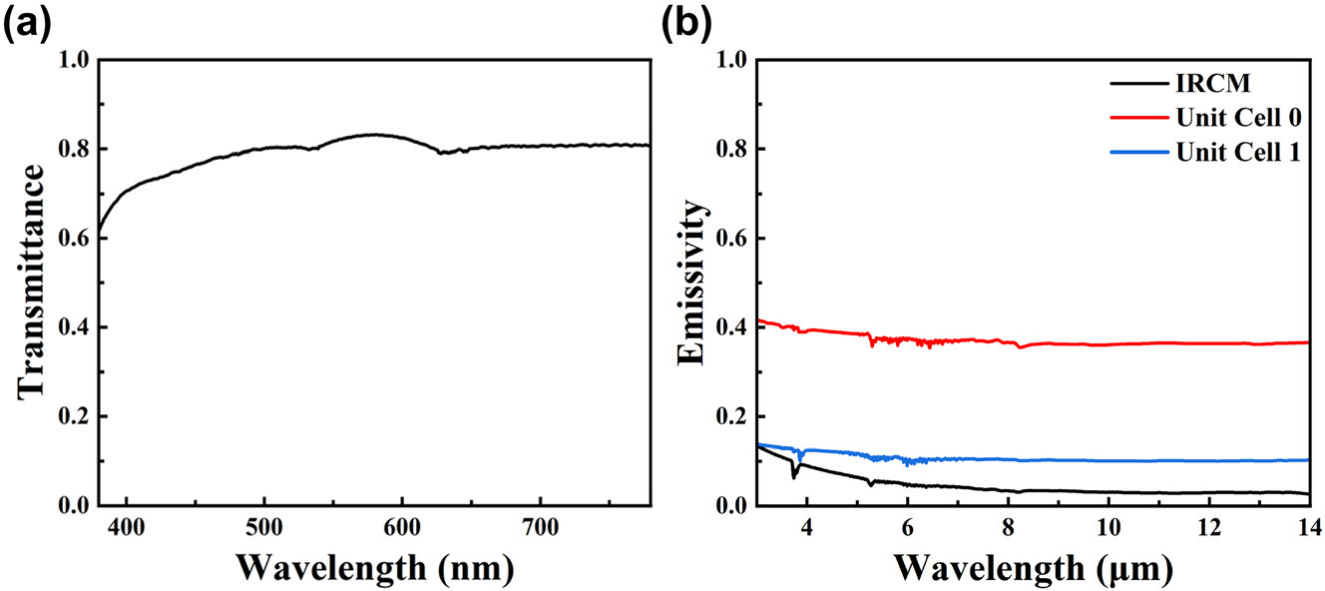
Measured result including (a) visible transmittance of each sample, (b) IR emissivity of each sample.

Figure 8a shows the measured optical transmittance of the IRCM. It was approximately 0.7. The measured results were inferior to the simulation results. This was because the upper and lower layers were sputtered with a multilayer film, whereas the calculated results involved only an upper film. The measured IR emissivity was approximately 0.05 in the 3–14 μm band as Figure 8b shows. This indicates that the prepared IRCM had a low IR emissivity. The emissivity was higher than the designed result at approximately 4 μm owing to the strong IR absorption of carbon–oxygen bonded structures in this band [62]. Moreover, each unit had a different IR emissivity. This could realize the IR camouflage performance.

**Figure 9:**
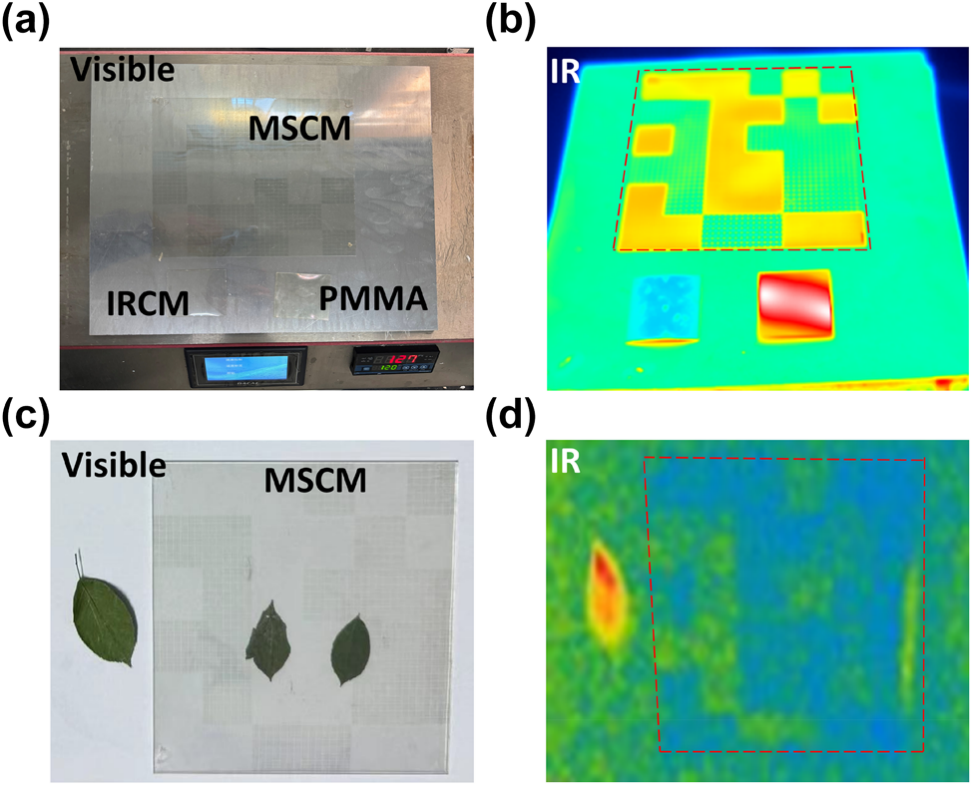
Measured result including (a). thermal imaging measurement setup and sample placement, (b) thermal image captured by thermal imager at 120 °C. (c) The actual application scenarios of the MSCM, (d) IR camouflage performance of the MSCM at room temperature.

To further verify the IR camouflage effect of the MSCM, the MSCM, IRCM, and PMMA were placed on the heating table simultaneously (Figure 9a). These were photographed by an IR thermal camera at 120 °C. As shown in Figure 9b, the MSCM camouflaged the IR radiation compared with the IRCM and PMMA. Moreover, the camouflaged IR radiation of the MSCM deceived the IR detection system, thereby achieving the effect of IR camouflage. The thermal images obtained at 50 °C and 90 °C are shown in Section S2.3 (Supporting information).

To better simulate actual application scenarios, three leaves were placed on the test panel, as shown in Figure 9c. Notably, the two leaves on the left side were covered with the MSCM. Evidently, all leaves were visible, and the MSCM did not affect their visibility characteristics. Figure 9d shows the leaves with different covers under an IR camera. The leaves on the right were detected conveniently, whereas the leaves enveloped by the MSCM disappeared.

**Figure 10:**
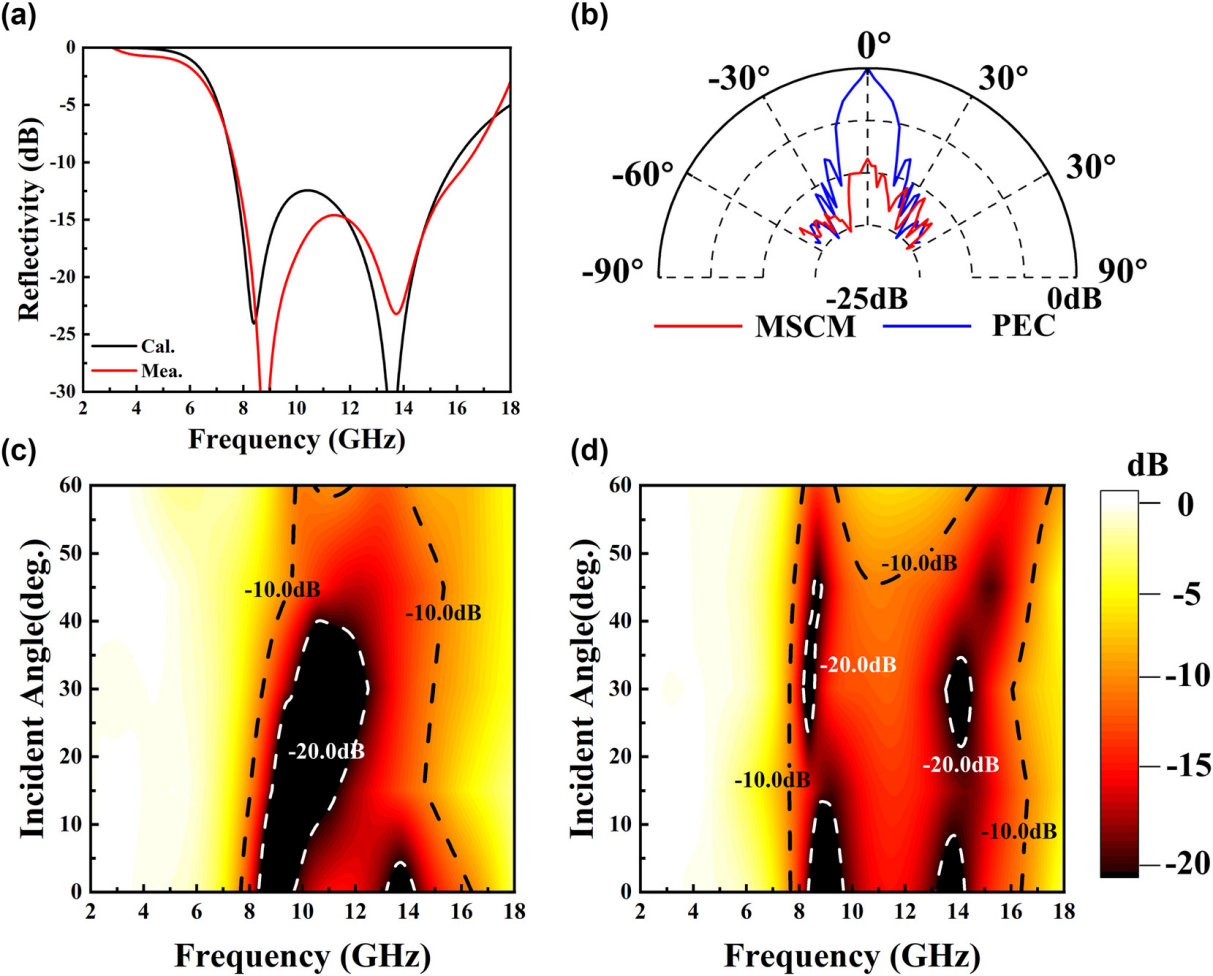
Measured reflectivity of the MSCM (a) under normal incidence, and the comparison between simulation results, (b) at different receiving angles. Measured reflectivity of the MSCM at different incident angles under (c) TE, (d) TM polarization mode.

Figure 10a shows the measured reflectivity of the MSCM under normal incident conditions. The −10 dB RCSR band is from 7.4 GHz to 16 GHz. The calculated and measured results are in good agreement. We can calculate the ultimate thickness corresponded to the microwave reflectivity to evaluate the RCS reduction more intuitively, which has the following form [63]:

(13) $$h_{\mathrm{roz}}=\frac{\overset{\infty }{\underset{0}{\int }}\ln\left|\rho \left(\lambda \right)\right|\text{d}\lambda }{2\pi^{2}}.$$

The calculated thickness corresponds to the reflectivity performance shown in Figure 10a is 2.672 mm, which is close to the thickness of the proposed structure (2.7 mm). Therefore, our design achieved the RCS reduction performance close to the ultimate one for a fixed thickness. Eq. (13) also suggests that increasing the total thickness of the metasurface structure could be a potential approach to achieve broader bandwidth microwave camouflage performance.

We also measure the reflectivity at different receiving angles under normal incidence conditions at 10 GHz. As shown in Figure 10b, there has a certain RCSR from 0° to 60°. Moreover, due to the limitation of the working range of the rocker arm in the arch system, our oblique incidence test can only reach 60°. To reveal the oblique incident stability, we measured the reflectivity of the MSCM at different incident angles, as shown in Figure 10c and d. It maintained a reflectivity less than −10 dB when the incident angle *θ* varied from 0° to 60° under both TE and TM polarization mode. However, the −10dB bandwidth was narrower with a larger *θ*. More information about the measured equipment and results are shown in Section S2.2 (Supporting information). Furthermore, considering real applications on curved targets, the proposed MSCM was enclosed at a certain angle with dimensions of 180 × 180 mm. More details about the curved MSCM are shown in Supporting Information. Most importantly, the MSCM displayed effective microwave camouflage at different incident angles under both TE and TM polarization modes.

**Figure 11:**
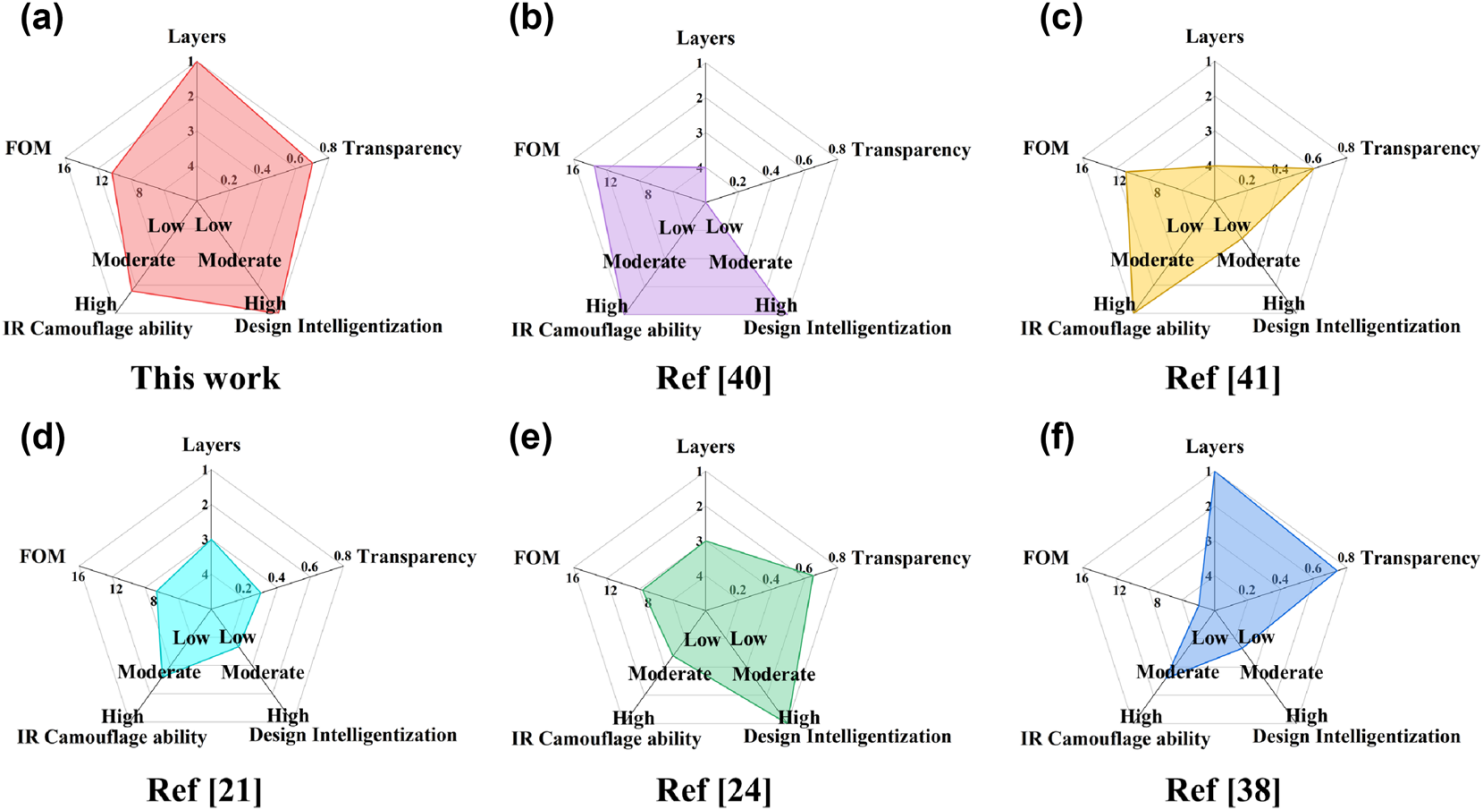
Comprehensive performance of the proposed and prevalent MSCMs across five dimensions. Here, the FOM can be calculated as follows, FOM = (c × Δf) / (h × f_0_ × f_L_). ∆f is the continuous bandwidth defined by a reflectivity below −10 dB. f_0_ and f_L_ denote the end and beginning of the working frequency, respectively. The IR camouflage capability is mainly aimed at detection from thermal imagers. Intelligent design implies the convenience of design methods.

Figure 11a–f presents the comprehensive performance of the proposed metasurface with those designed in other related works across five dimensions. Those five dimensions are the layer number, camouflage capability in the IR band, figure of merit (FOM) in the microwave band, visible transparency, and intelligent design method, respectively. As illustrated, the proposed MSCM exhibits significant advancements in multispectral camouflage performance compared to existing studies. It demonstrates comprehensive superiority, particularly in terms of infrared and microwave camouflage capabilities, while maintaining a single-layered structure and high optical transparency.

## 4 Conclusions

In this study, a cross-scaled MSCM was designed and verified. First, an IRCM based on multilayered films was designed to achieve both high visible transmittance and low IR emissivity. Then, one side of the IRCM was processed into a diffusion metasurface to achieve microwave camouflage. Owing to the high electrical conductivity of the back IRCM, almost none of the incident microwaves could be transmitted, and over 80 % of the incident microwaves ranging from 7 GHz to 16 GHz diffused in different directions. Moreover, the MSCM maintained a certain oblique incident stability for microwaves. Finally, the MSCM was heated up to 120 °C. The thermal image shows the IR camouflage performance with two IR radiation intensities. Although single metasurface was present, it displayed multispectral camouflage performance in the visible, IR, and microwave bands. The entire design and optimization process were assisted by AI, which saved a significant amount of resources and helped us obtain the best results.

## Supplementary Material

Supplementary Material Details
