# Supplementary material for: Bio-inspired multispectral camouflage material for microwave, infrared, and visible bands based on single hierarchical metasurface
Source: Nanophotonics. 2025 May 1;14(12):2173–86. doi: 10.1515/nanoph-2025-0024 (PMC12147556; doi:10.1515/nanoph-2025-0024)
Supplement: Supplementary file 1 — Supplementary Material Details [file j_nanoph-2025-0024_suppl_001.docx]

Supporting Information

**Section S1. Related informations in modelling, design and optimization**

Section S1.1 ECM of the multi-layer films and EM parameters of Ag and AZO

The schematic model and ECM model of the oxide and metal overlapping multilayer film are shown in Figure S1.


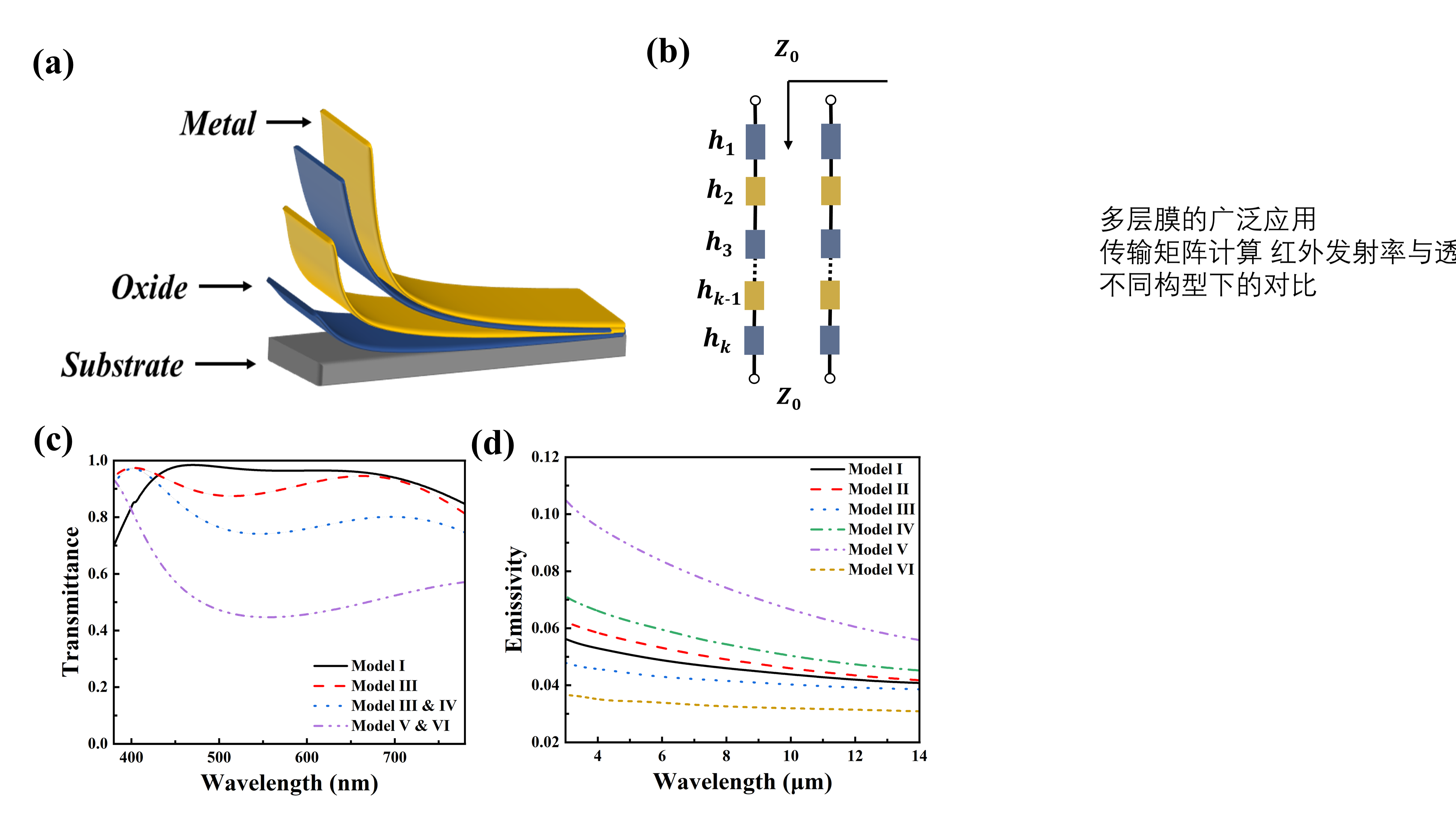


**Figure S1.** (a) schematic of multi-layer films, (b) ECM of the multi-layer films.

Figure S2 (a) to (d) plot the complex refractive index and dielectric constant of Ag and AZO ranging from 3μm to 14μm [1-2]and 300nm to 800nm [3-4]. The parameters are calculated through the following equation[5],

| $\varepsilon^{,}=k^{2}-n^{2},$ | (S1) |
| --- | --- |
| $\varepsilon^{,,}=2\times k\times n,$ | (S2) |

where n and k represent the refractive index and extinction coefficient, respectivitly.


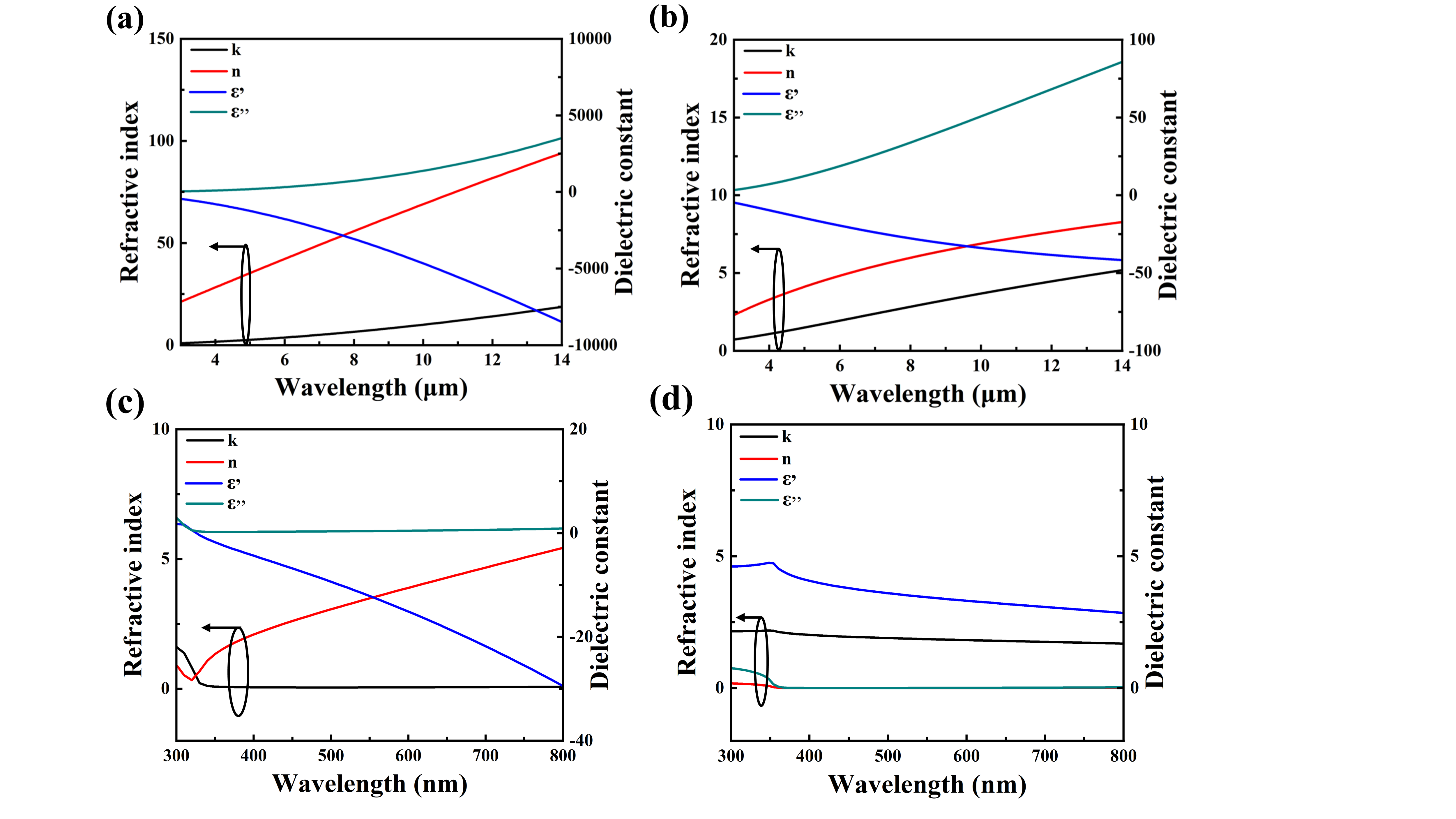


**Figure S2.** Refractive index and dielectric constant of (a) Ag, (b) AZO ranging from 3-14μm. Refractive index and dielectric constant (c) of Ag, (d) AZO ranging from 300-800nm.

Section S1.2 Influence of the PMMA substrate on the IR emissivity and visible transmittance

Figure S3 (a) plots the refractive index and dielectric constant calculated through Eq(S1) of PMMA[6]. In this band, the extinction coefficient k equals 0, which means no loss occurs, which is also confirmed by the calculated transmittance shown in Figure S3 (b), the transmittance is 100%. Figure S3 (c) plots transmittance of of Model I in IR band, the transmittance is almost 0%. Generallty, the influence of the PMMA substrate on the IR emissivity and visible transmittance can be neglected.


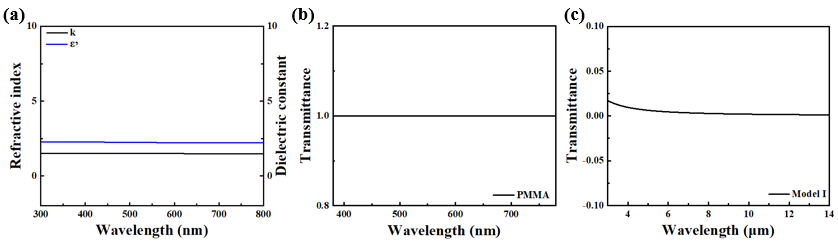


**Figure S3.** (a) Refractive index and dielectric constant of PMMA ranging from 300-800nm, (b). transmittance of PMMA ranging from 300-800nm (c), transmittance of Model I ranging from 3-14μm.

Section S1.3 Normalized magnetic field distributions of Model I to VI.

As Figure S4 shows, the high magnetic field is concentrated in the middle Ag layer of Model I, II and III. However, in Model V, there is no such phenomenon.


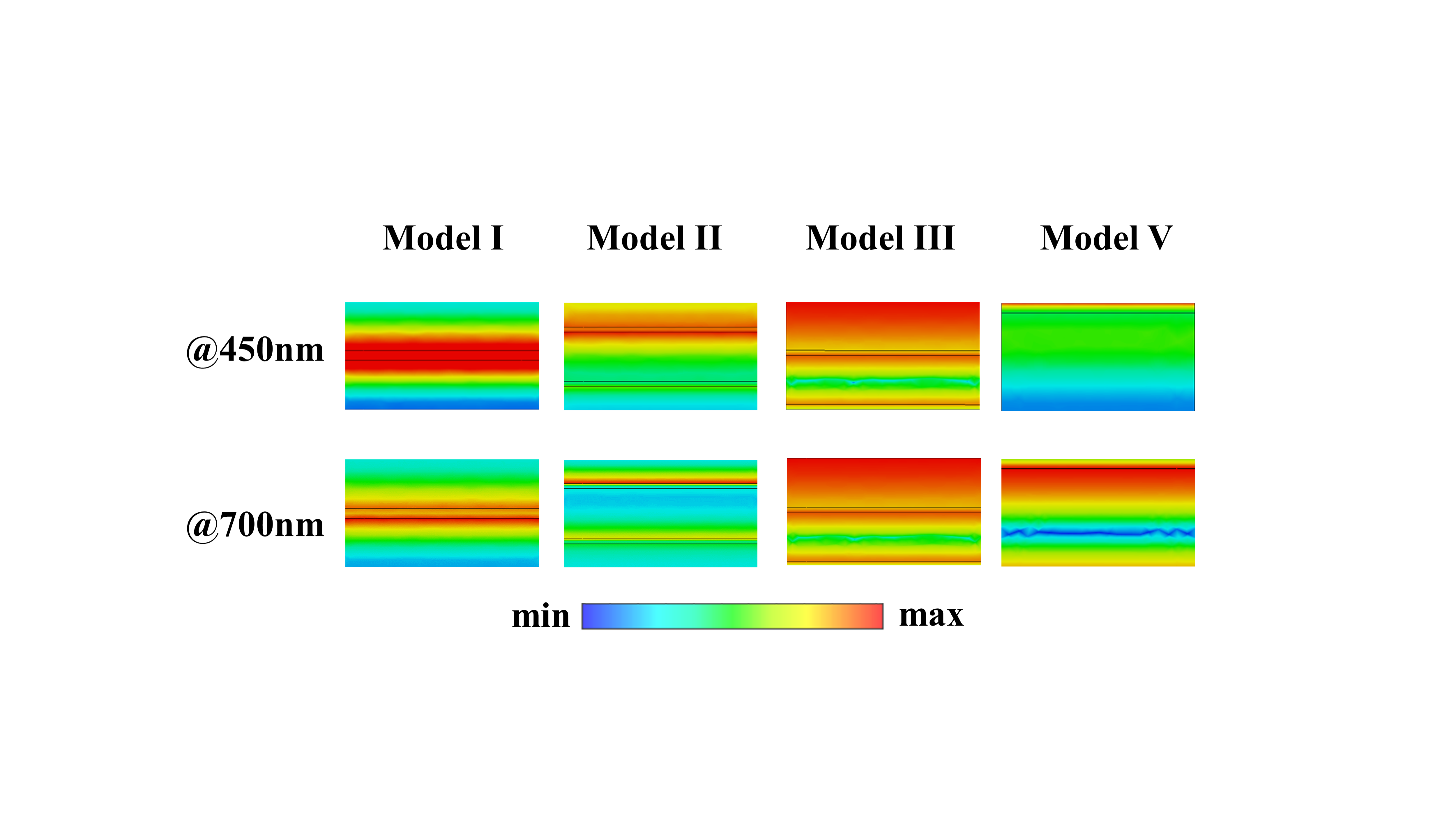


**Figure S4**. Normalized magnetic field distributions at 450 nm and 700 nm of Model I, II, III and V.

As Figure S5 shows, in Model I, II, III and VI, the incident wave is almost reflected by the upper layer, while in Model IV and V, neither high reflection nor high transmission happened.


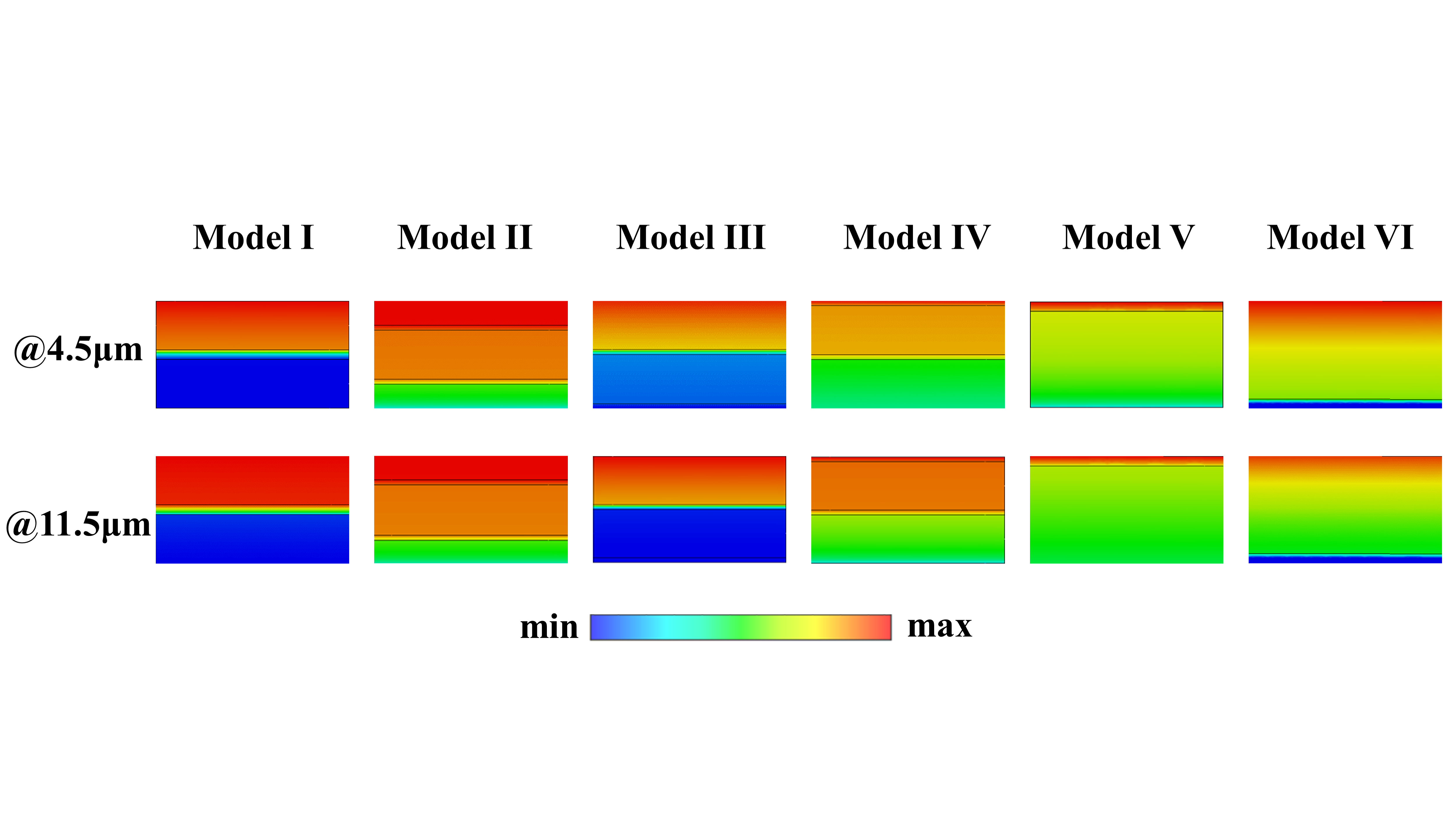


**Figure S5.** Normalized magnetic field distributions at 4.5μm and 11.5μm of Model I to VI.

Section S1.4 Parameter sweeping results of the film

Figure S6 (a) and (b) shows the parameter sweeping results of Model I. Firstly, we change the thickness of the oxide, as shown, due to the reduction of thickness, the transmittance is improved, so does the IR emissivity.


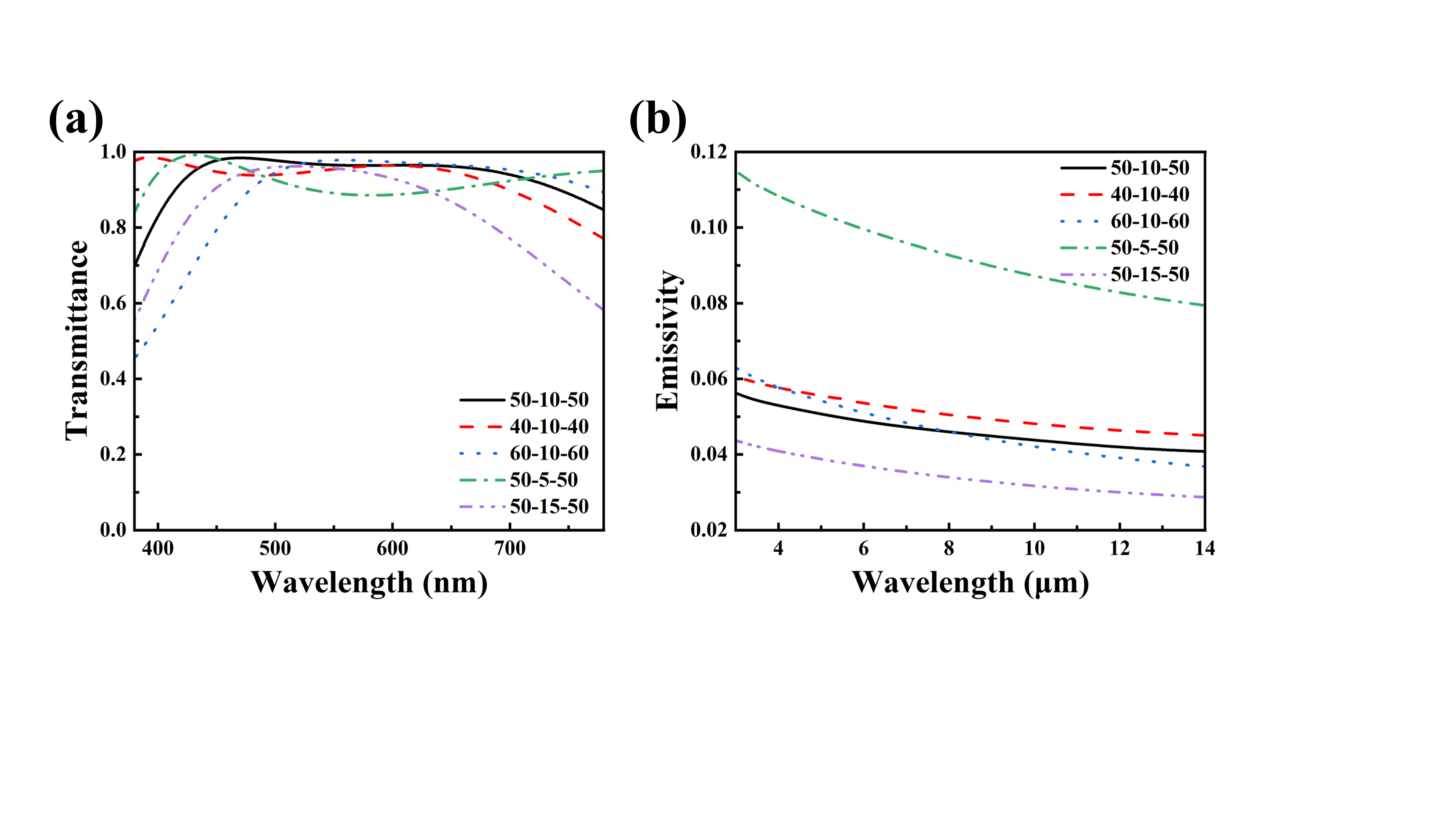


**Figure S6.** Parameter sweeping results in (a). visible and (b).IR bands.

Then, we change the thickness of the metal, as shown, due to the reduction of thickness, the transmittance is improved, as does the IR emissivity. It seems that the IR emissivity and visible transmittance change as thickness changes. However, blindly parameter sweeping is time-consuming and inefficient.

Section S1.5 Calculation process of equivalent parameters

Figure S7 shows the ECM of the microwave diffusion unit cell.


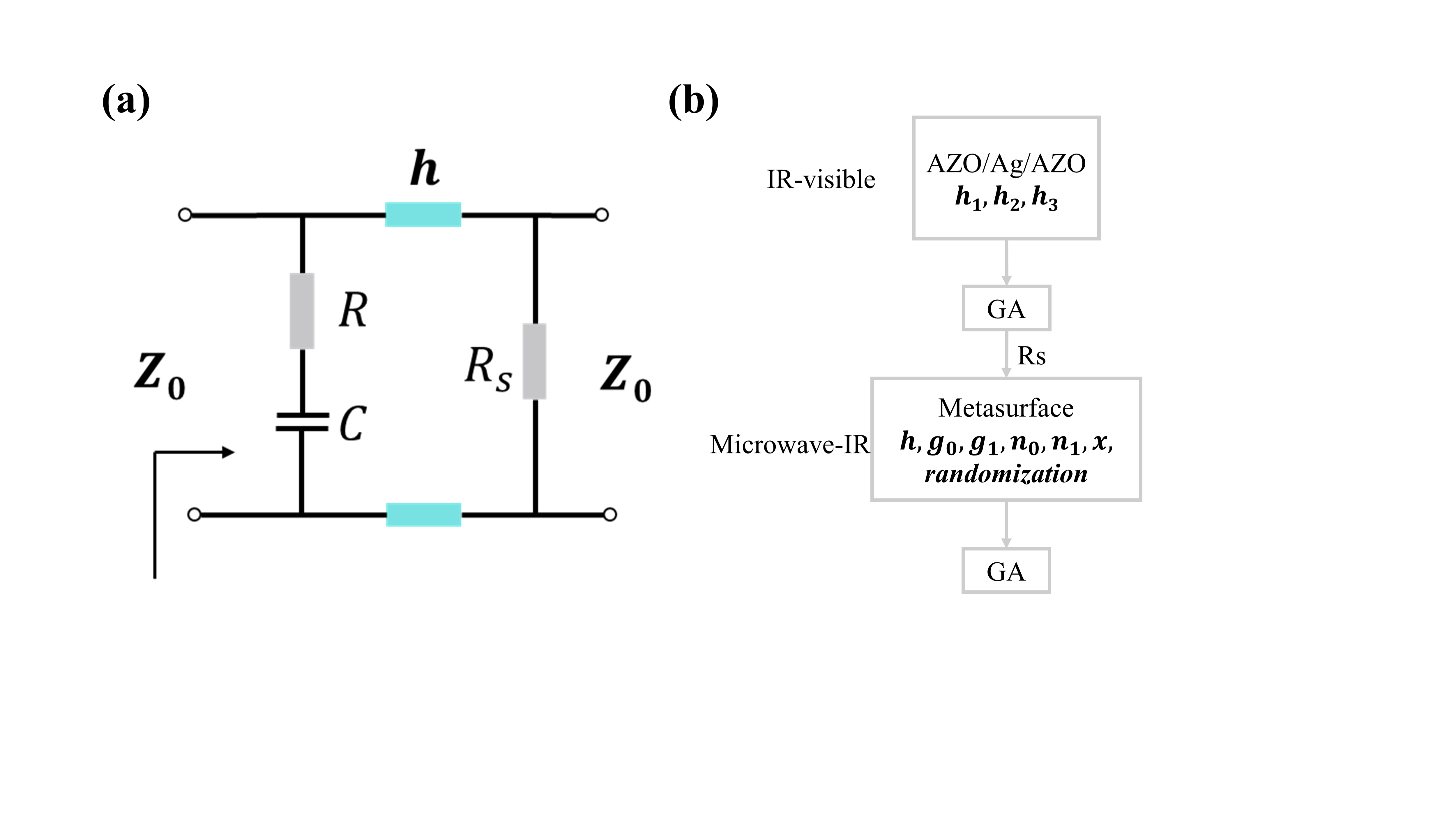


**Figure S7.** ECM of the unit cell

The calculation process of equivalent parameters are shown as follows[7],

| $p_{k}=\frac{p}{n_{k}},$ | (S3) |
| --- | --- |
| $R_{k}=R_{s}\left( \frac{p_{k}}{p_{k}-g_{k}} \right)^{2},$ | (S4) |
| $C_{k}=4\varepsilon_{eff}Y_{0}\frac{p_{k}-g_{k}}{p_{k}}F\left( p_{k},g_{k},\lambda,\theta\right),$ | (S5) |
| $F\left( p_{k},g_{k},\lambda,\theta\right)=\frac{p}{\lambda}\cos\theta\left\{ ln\left[ \csc\left( \frac{\pi g_{k}}{2p_{k}} \right) \right]+G{(p}_{k},g_{k},\lambda,\theta) \right\},$ | (S6) |
| $\begin{aligned} G\left( p_{k},g_{k},\lambda,\theta\right)=\frac{0.5\left( 1-b^{2} \right)^{2}\left[ \left( 1-\frac{b^{2}}{4} \right)\left( a_{+}+a_{-} \right)+4b^{2}a_{+}a_{-} \right]}{1-\frac{b^{2}}{4}+b^{2}\left( 1+\frac{b^{2}}{2}-\frac{b^{4}}{8} \right)\left( a_{+}+a_{-} \right)+2b^{6}a_{+}a_{-}}, \end{aligned}$ | (S7) |
| $a_{\pm}=\frac{1}{\sqrt{1\pm\frac{2p\sin\theta}{\lambda}-\left( \frac{p\cos\theta}{\lambda} \right)^{2}}}-1,$ | (S8) |
| $b=\sin\frac{\pi g}{2p},$ | (S9) |
| $\varepsilon_{eff}=\frac{\left( \varepsilon_{PMMA}+1 \right)}{2},$ | (S10) |

where *θ* is the incident angle, *p_k_* and *g_k_* are structure parameters shown in Figure 2 (a), *k* equals to 0 and 1, representing each unit cell.

Section.1.6 Related information about GA optimization

The complete flow chart of GA program as shown in Figure S8. The GA was incorporated with the ECM to optimize the structure parameters in the ranges shown in Table S1 Row 3. The setting of GA is shown in Table S2.


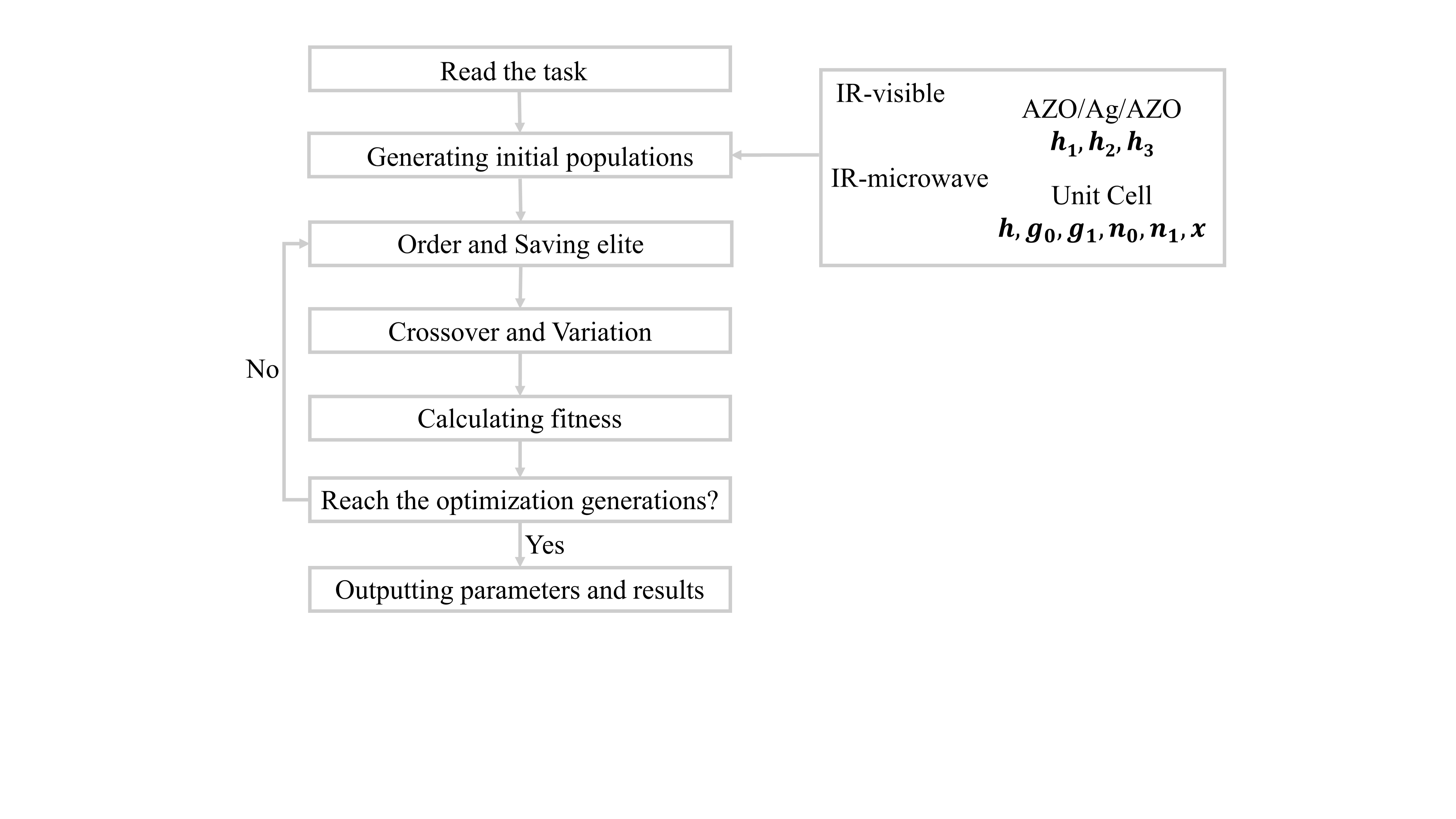


**Figure S8.** Designed flowchart of the GA optimization

The detailed steps are as follows:

Step (1) Reading the task,

Step (2) Generating random initial populations,

Step (3) Encoding the populations into binary chromosomes based on the population number and DNA population size settings,

Step (4) Performing mutation and crossover on binary chromosomes according to the mutation and crossover probability settings,

Step (5) Decoding the mutated and crossed chromosomes into populations,

Step (6) Selecting elite individuals and checking whether the generation limits have been reached. If not, return to step (3),

Step (7) Outputting the parameters and results.

Concerning the fitness function, we divided both the IR band ranging from 3μm to 14μm and visible band ranging from 380nm to 780nm into 101 points. Parameters including h1, h2 and h3 can be optimized and the fitness F1 is defined as,

| $F_{1}=\frac{0.5\sum_{u=0}^{100} \left\{ \begin{aligned} 1 &T\left[ u \right]\geq0.8 \\ 0 &T\left[ u \right]<0.8 \end{aligned} \right.+0.5\sum_{v=0}^{100} \left\{ \begin{aligned} 1 &E\left[ v \right]\leq0.1 \\ 0 &E\left[ v \right]>0.1 \end{aligned} \right.}{101},$ | (S11) |
| --- | --- |

where $T\left[ u \right]$ represent the transmittance at the *u*th wavelength point,$E\left[ v \right]$ represent the emissivity at the *v*th wavelength point. Since we want to obtain a high visible transmittance and low IR emissivity film, the weights are split in half.

Similarly, we divided the microwave band from 2-18GHz into 101 points, and the fitness value of each frequency point was 1. The normalized fitness *F_2_* is defined as,

| $F_{2}=\frac{\sum_{w=0}^{100} \left\{ \begin{aligned} 1 &\Gamma\left[ w \right]\leq-10dB \\ 0 &\Gamma\left[ w \right]>-10dB \end{aligned} \right.}{101},$ | (S12) |
| --- | --- |

where $\Gamma\left[ w \right]$ represent the reflectivity at the *w*th frequency point. The total fitness$F_{3}$ is defined as follows,

| $F_{3}={F_{1}+F}_{2}$ | (S13) |
| --- | --- |

The final fitness is shown in Figure S9. After completing the whole optimization process, the structure parameters are shown in Table S1 Row 4. To simplify the process, we adjusted the parameters as listed in the third row of Table S1 Row 5. The whole optimization process costs 23 minutes.

**Table S1.** Ranges and Results of the GA process.

| Results | Multilayered film | | | Unit cell | | | | | | | |
| --- | --- | --- | --- | --- | --- | --- | --- | --- | --- | --- | --- |
|  | h_1_(nm) | h_2_(nm) | h_3_(nm) | p(mm) | h(mm) | g_0_(mm) | g_1_(mm) | n_0_ | n_1_ | x | Rs(Ω/sq) |
| Ranges | 30-60 | 8-16 | 30-60 | 30 | 1-3 | 0.1-0.25 | 0.05-0.25 | 15-50 | 3-10 | 0-1 | 1.1 |
| GA | 50.36 | 11.93 | 48.87 |  | 2.724 | 0.138 | 0.208 | 46.21 | 6.78 | 0.523 |  |
| Processed | 50 | 12 | 50 |  | 2.7 | 0.14 | 0.21 | 46 | 7 | 0.5 |  |

The components in the ECM regarding to the proposed structural parameters are as follows: R_0_ = 1.62Ω, R_1_ = 1.1Ω, C_0_ = 0.117pF, C_1_ = 0.0077pF.

**Table S2.** Settings of the GA process.

| DNA population size | 10 |
| --- | --- |
| Population number | 400 |
| Crossover probability | 0.01 |
| Variation probability | 0.8 |
| Optimization generation | 1000 |


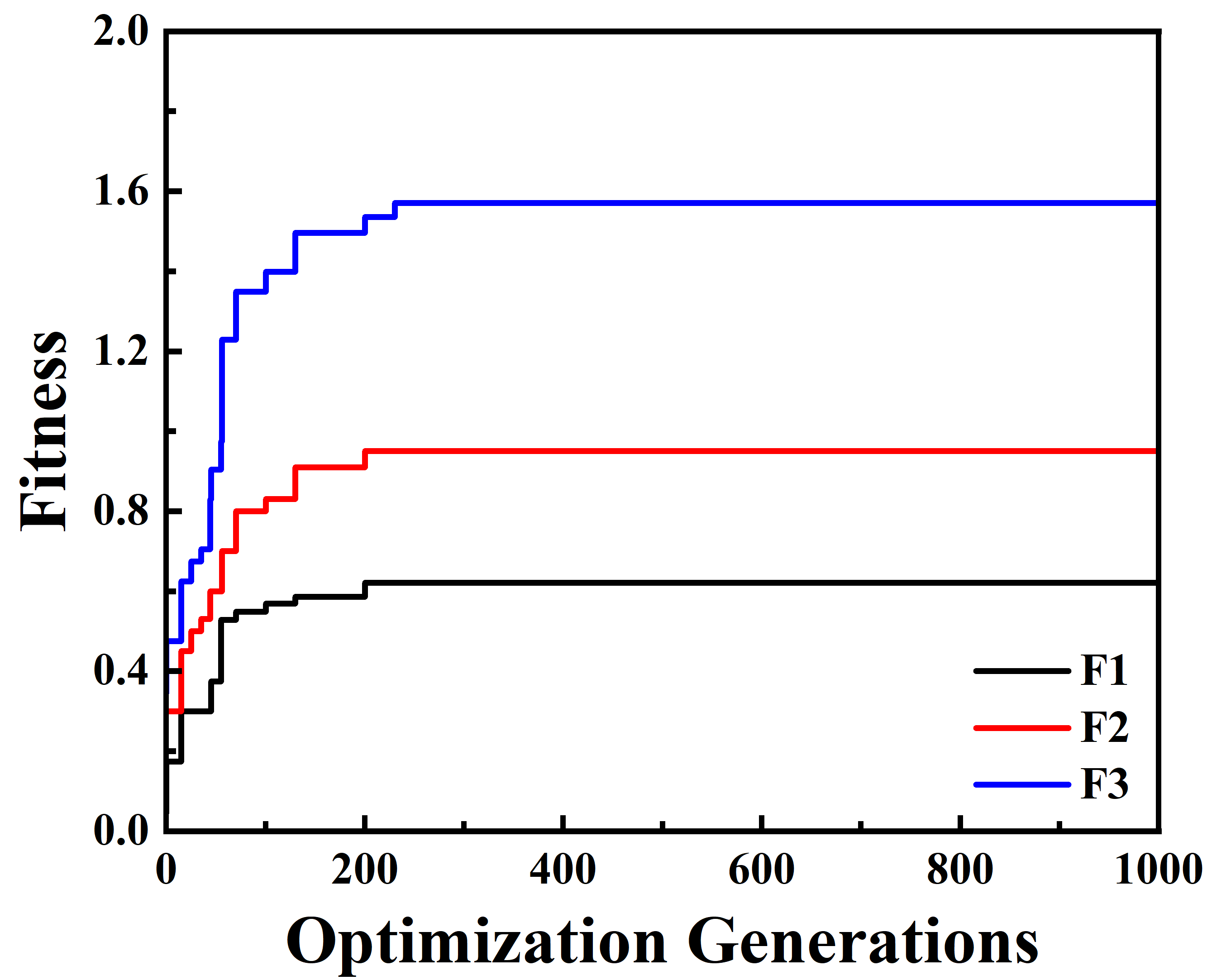


**Figure S9.** Fitness of GA corresponded to optimization generations.

The E of the film is shown in Figure S10 (a) which is smaller than 0.1 from 3μm to 14μm. The T of the film is shown in Figure S10 (b) which is bigger than 0.8 from 380nm to 780nm. Full-wave simulation was carried to verify the optimization results as shown in Figure S10 (a) and (b), which is in a good agreement with the calculation result. The simulation is carried through CST Studio Suite 2021 using Frequency Domain Solver.


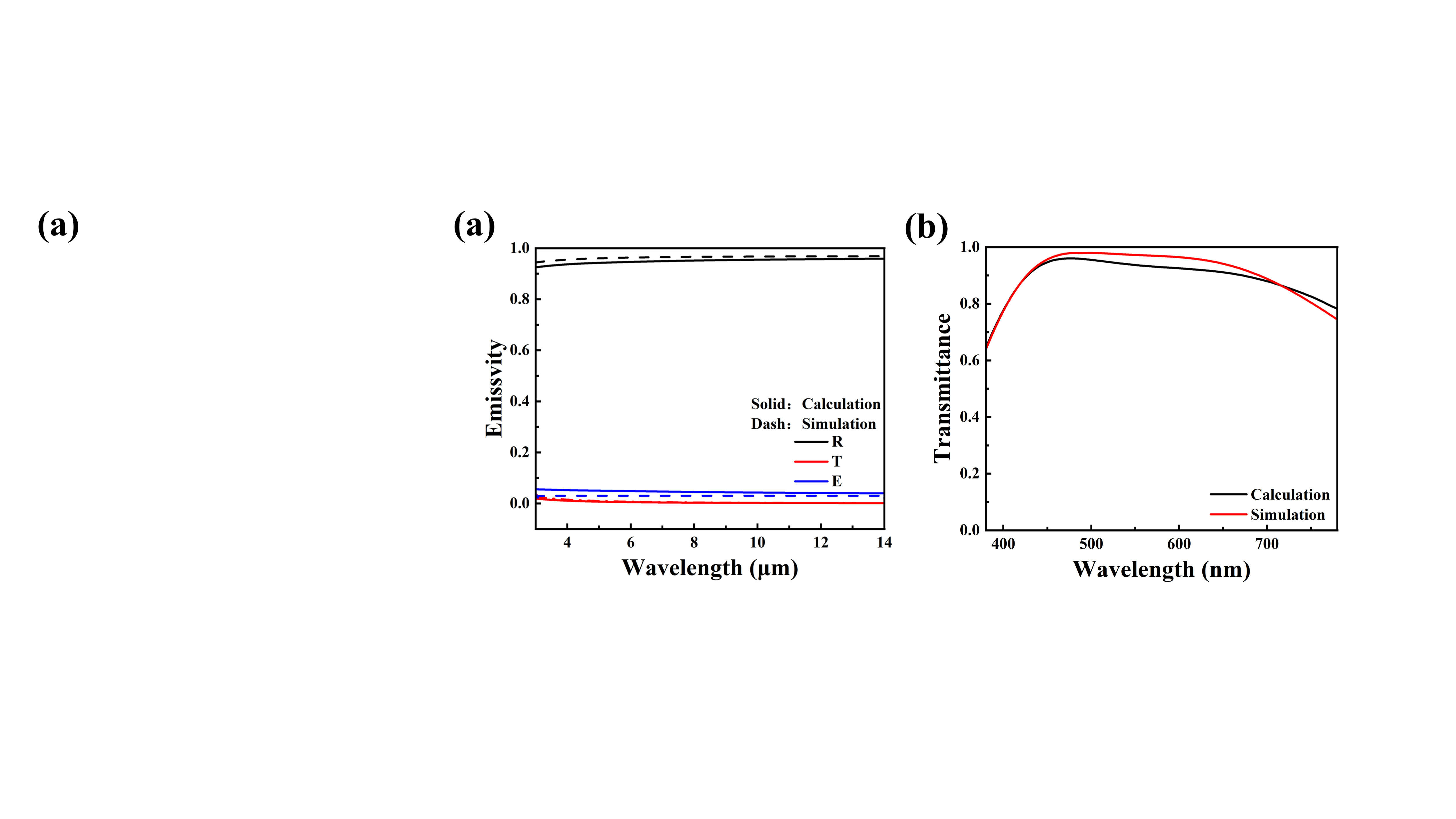


**Figure S10.** (a) E, R, T of film from 3μm to 14μm (b) T of film from 380nm to 780nm.

Section S1.7 Working principles of microwave camouflage

Figure S11 (a) plots the arrangement of the MSCM. Figure S11 (b) plots the simulated RCS of IRCM and MSCM, clearly, the RCS has a noticeable reduction. Figure S11 (C) to (d) plot the calculated transmission amplitude and phase of Unit 0 and 1.


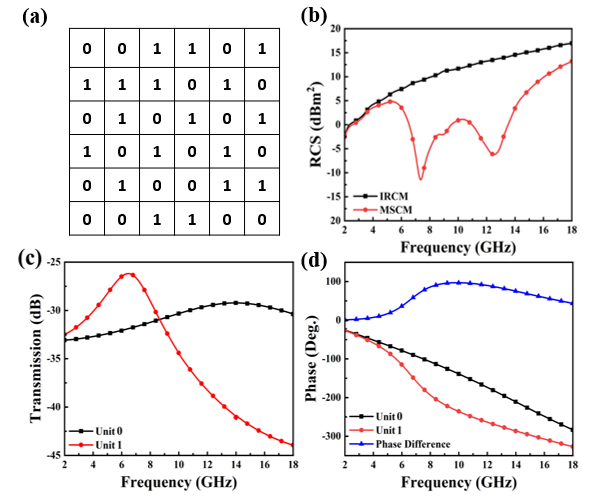


**Figure S11.** (a) Arrangement of the MSCM, (b) RCS of IRCM and MSCM,

(c) transmission amplitude and (d) phase of Unit 0 and 1.

The total energy distribution is calculated as follows[8], Figure S12 shows the energy distribution, clearly, diffusion plays a major role.

| $\left\{ \begin{aligned} \left\vert{x\times A}_{0}\exp\left( jP_{A_{0}} \right)+\left( 1-x \right)\times A_{1}\exp\left( jP_{A_{1}} \right) \right\vert^{2} \mathrm{reflection} \\ \left\vert{x\times T}_{0}\exp\left( jP_{T_{0}} \right)+\left( 1-x \right)\times T_{1}\exp\left( jP_{T_{1}} \right) \right\vert^{2} \mathrm{transmission} \\ 1-\left\vert{x\times A}_{0}+\left( 1-x \right)\times A_{1} \right\vert^{2}-\left\vert{x\times T}_{0}+\left( 1-x \right)\times T_{1} \right\vert^{2} \mathrm{absorption} \\ 1-\mathrm{reflection}-\mathrm{transmission}-\mathrm{absorption} \mathrm{diffusion} \end{aligned}, \right.$ | (S15) |
| --- | --- |


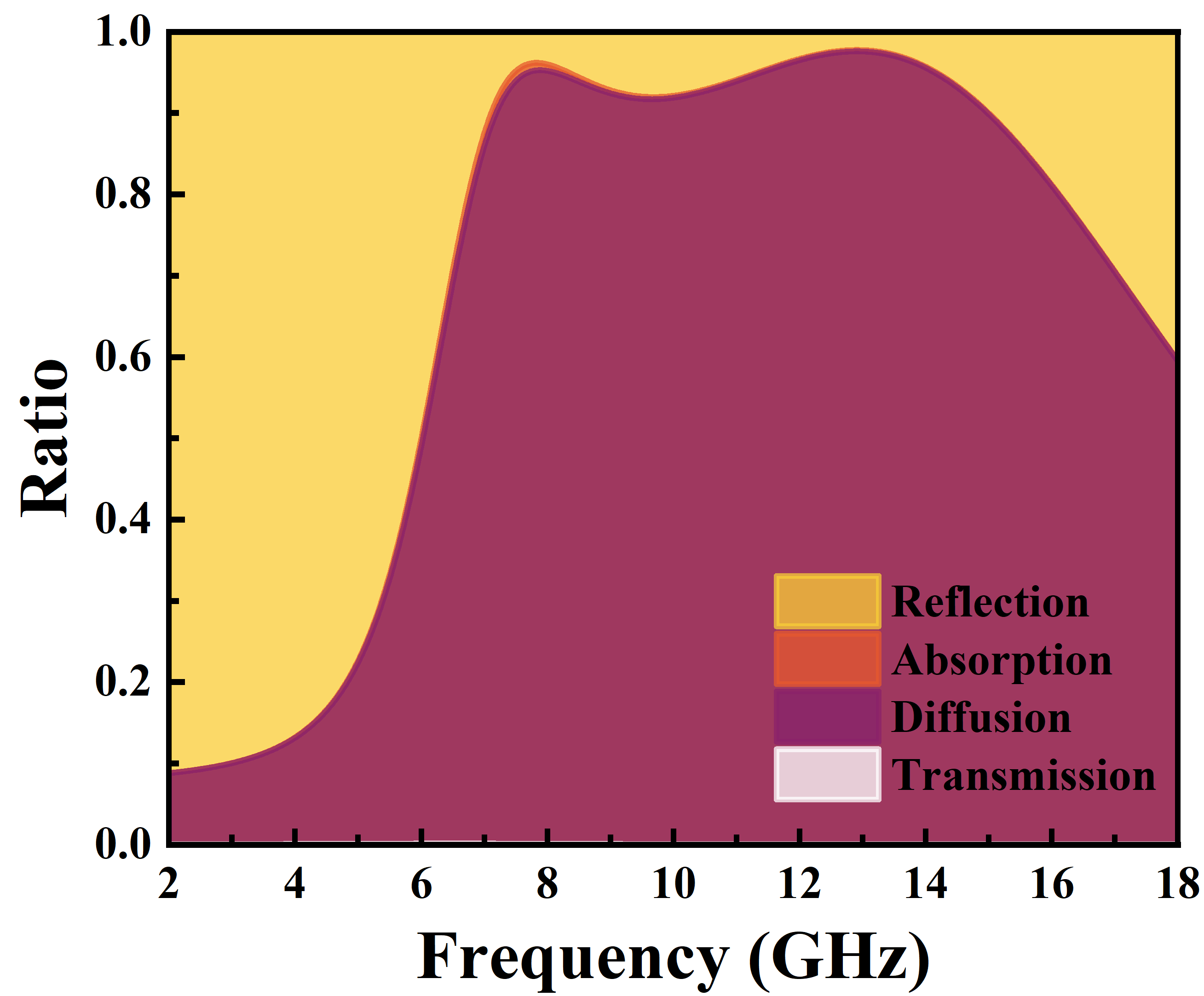


**Figure S12.** Energy distribution of the MSCM ranging from 2-18GHz.

Section S1.8 Far-field scattering situation


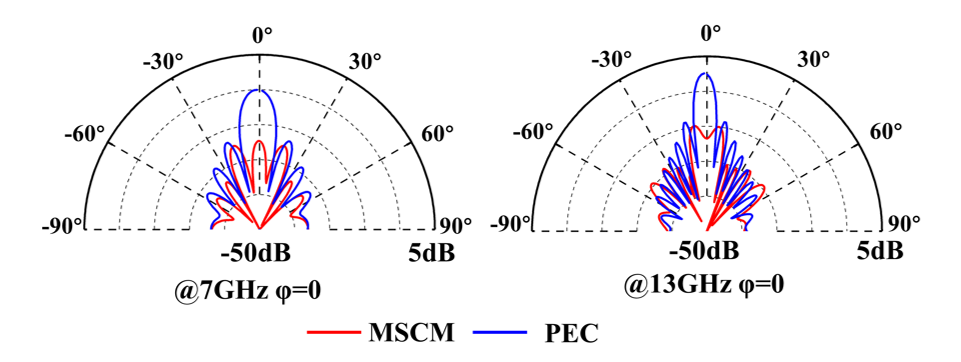


**Figure S13.** 1D far-field scattering pattern of MSCM and PEC at7GHz, φ=0, 13GHz, φ=0.

Figure S13 shows the 1D far-field scattering pattern of the MSCM and PEC for azimuth angle φ=0 at 7GHz, 13GHz, respectively. It has a significant RCS reduction for the backward lobe.


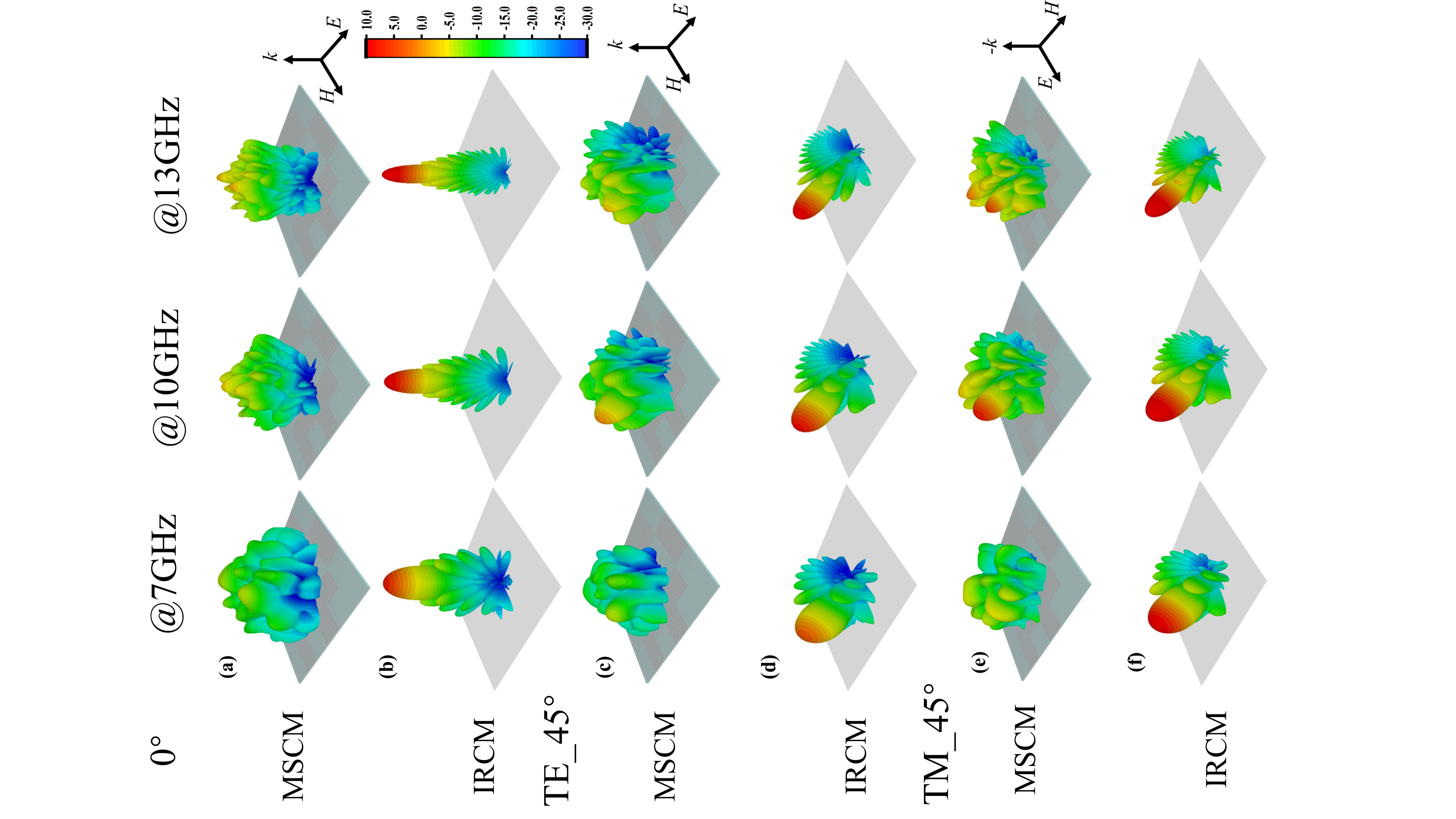


**Figure S14.** 3D far-field scattering under the normal incidence of (a) MSCM (b) IRCM at 7GHz, 10GHz, 13GHz, under the TE polarization incidence of (c) MSCM (d) IRCM at 7GHz, 10GHz, 13GHz, under the TM polarization incidence of (e) MSCM (f) IRCM at 7GHz, 10GHz, 13GHz.

Different scattering mechanisms at different frequencies can be figured out from the far-field scattering patterns. The three dimensional (3D) far-field scattering patterns under the normal illumination of a plane wave are shown in Figure S14 (a). It clearly presents that the scattered wave from the MSCM are randomly spread into numerous directions in the whole upper half-space at 7GHz, 10GHz and 13GHz under normal incidence, indicating broadband performances. In contrast, the 3D far-field result of the back IRCM only has a dominating specular reflection with ultralow side-lobes, as shown in Figure S14 (b). The working mechanism can also be extended to oblique incident cases as shown in Figure S14 (c) to (f), where the MSCM works well under the EM waves with 45° incidence angle. Similar diffusion-like scattering can also be found under both transverse electric (TE) and transverse magnetic (TM) polarization incidence or at other frequencies during the working band. The RCS reduction performance for TM polarization is much better than that for TE polarization at 15 GHz. The disparity between performances for TE and TM polarization is reasonable since the EM responses of stereo meta-atoms are spatial dispersive[9].

The 3D far-field scattering patterns under the normal illumination of a plane wave are shown in Figure S15 (a), the scattered wave from the curved MSCM are randomly spread into numerous directions in the whole upper half-space at 7GHz, 10GHz and 13GHz under normal incidence, indicating broadband performances. Also, the curved IRCM only has a dominating specular reflection with ultralow side-lobes, as shown in Figure S15 (b). As shown in Figure S15 (c) to (f), where the MSCM works well under the EM waves with 45° incidence angle. Similar diffusion-like scattering can also be found under both TE and TM polarization incidence or at other frequencies during the working band, which means the MSCM would have more applications for curved surfaces.


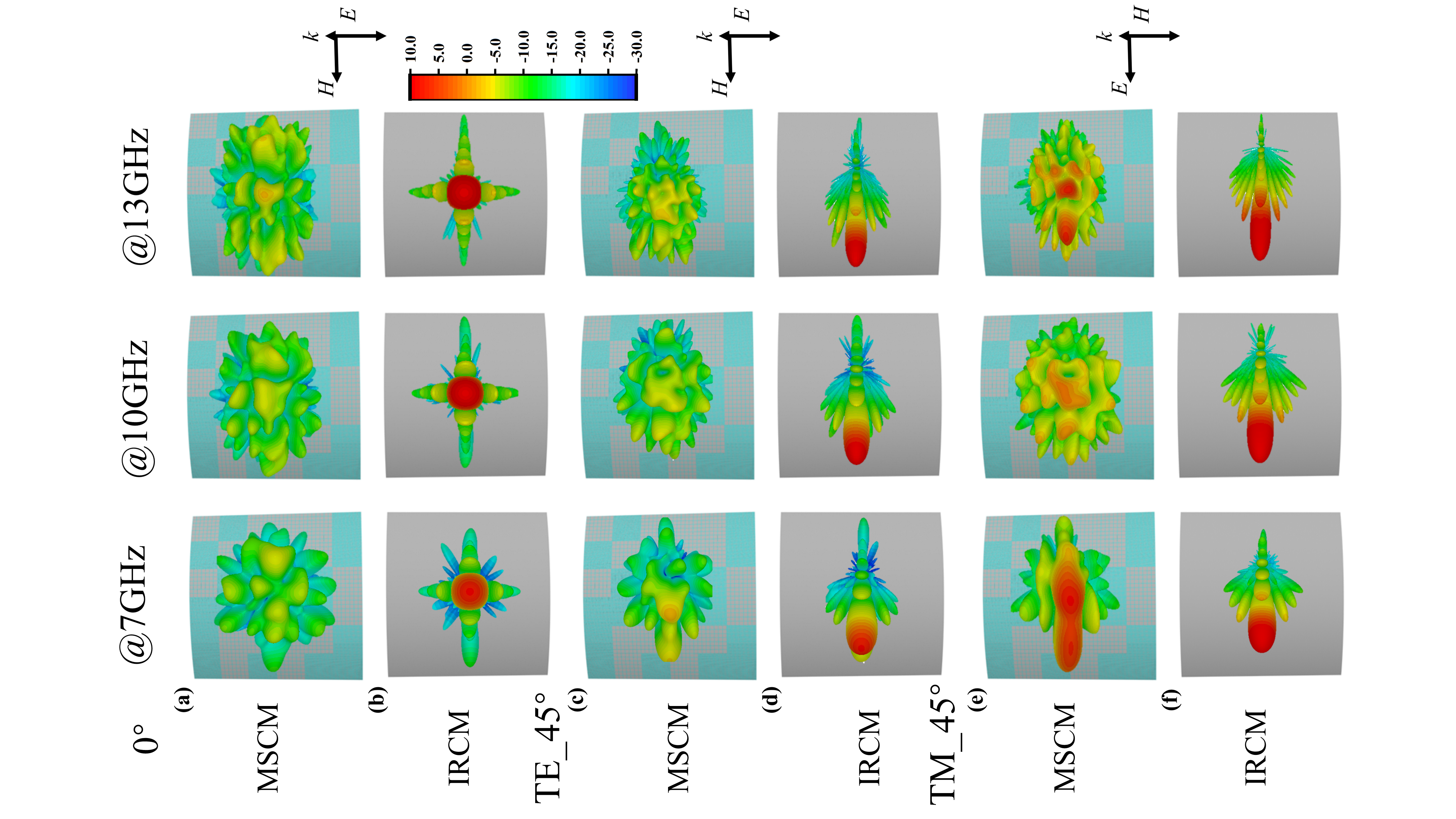


**Figure S15.** 3D far-field scattering under the normal incidence of (a) curved MSCM (b) curved IRCM at 7GHz, 10GHz, 13GHz, under the TE polarization incidence of (c) curved MSCM (d) curved IRCM at 7GHz, 10GHz, 13GHz, under the TM polarization incidence of (e) curved MSCM (f) curved IRCM at 7GHz, 10GHz, 13GHz.

**Section S2. Experimental Section**

Section S2.1. Fabrication equipment

Sample was prepared by magnetron sputtering technology according to the optimized thickness on the both sides of a 2.7 mm-thick double polished PMMA. First, the PMMA substrate was cleaned by ultrasonic wave with acetone and isopropanol for 10min and with deionized water for 10min, and it was dried with high-pressure nitrogen. In this work, YFX-CK3B-400A (Reborn Tech.) magnetron sputtering coater shown in Figure S16 was used to fabricate the AZO/Ag/AZO film. The coating power was set as 300W and the air pressure was 3m Torr. The time for each layer is 310s, 7s, 320s, respectively. The substrate was evaporated with Ag target and AZO target respectively for 15 min. After finish the one-side sputtering, we took the sample and flip to the other side, when the temperature returns to room temperature, the sputtering process was repeated. Finally, the double-sides IRCM is well done.


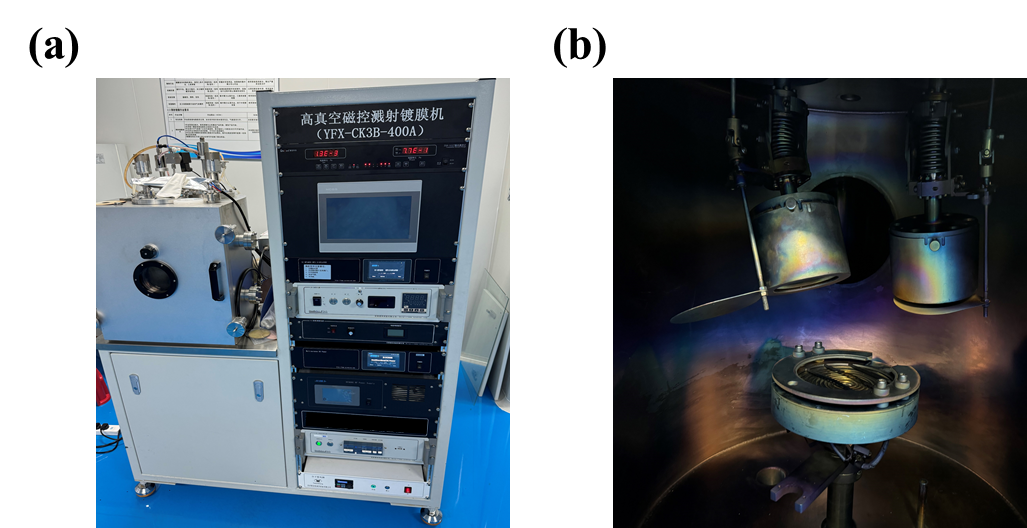


**Figure S16** (a). YFX-CK3B-400A magnetron sputtering coater and (b) the working cavity

The AZO (ZnO: Al_2_O_3_= 98:2wt%) and Ag target used in the process is from Fuzhou Innovation optoelectronic Technology Co., Ltd with a purity of 99.999% and 99.9999%, respectively. One side of the sample is processed by nanosecond lasers SK-CX20 (Shanghai Sanko Laser Technology Co.) according to the optimized result. The wavelength of laser is 1064nm, the output power is 20W.

Section S2.2. Measured equipment and results

Square resistance is measured by the HPS2523 Square Resistance Tester (Helpass, Co. Ltd) shown in Figure S17 (a). Digital photographs of the samples were taken using the back camera of an iPhone 15 ProMax. The cold field emission scanning electron microscope (SEM) is measured by SU8220 (Hitachi Limited). The IR emissivity is measured by Hyperion 2000 Fourier transform IR microscope (Bruker Corporation) shown in Figure S17 (b). The optical transmittance is measured through SolidSpec-3600 DUV (Shimadzu Corporation).

The IR thermal picture is photted by the IR thermal imager TESTO 885 (Testo AG) and the heating plate is V-D100. (Dongguan JFTOOIS Co., Ltd.). The IR dector is FLIR E4(Teledyne FLIR). The IR test is carried out at 14:00 July 15th 2024, Nanjing, China. The outside temperature is 39°C and the indoor temperature is 27°C. Instantaneous Field of View (IFOV) is a vital factor of the thermal infrared imager. The thermal infrared imager (TESTO-885, Testo AG) has an IFOV of 1.13mrad, and the distance d between the lens and the heating table is about 60 cm. Therefore, the minimum detection size is d× IFOV=0.678mm, which means the image detail smaller than 0.678mm could not be recorded by the imager.

The arch system for microwave measurement has a vector network analyzer (VNA) E8363A (Agilent Technologies Co., Ltd), a pair of broadband double-ridged horn antenna (1GHz to 18GHz) and a pair of power-driven antenna hangers for oblique incidence measurement.

Unlike the conventional NRL Arch test system, our two antenna mounts are motorized and can be controlled independently and asynchronously. To measure the bistatic RCS, we first measured the maximum reflectivity of the PEC plate under normal incidence of electromagnetic waves as the reference value. Then, we fixed one antenna and rotated the other antenna to a specific position on the rail to measure the reflectivity of the MSCM at that angle. Then, by normalizing the measured reflectivity with the maximum reflectivity of the metal plate, we can obtain the bistatic RCS as shown in Figure 10(b).

Section S2.3. Related measured results

As Figure S17 (a) shows, clearly, the prepared IRCM has a certain optical transmittance and the measured the square resistance is 1.01Ω/sq. Figure S17 (c) shows the measured IR emissivity of 2.7mm PMMA medium, the medium has more than 0.9 emissivity in IR band. Figure S17 (d) shows the overall pattern of the MSCM under SEM, the structure parameters are in a suitable range. Figure S17 (e) shows the arch system for microwave measurement and Figure S17 (f) shows reflectivity at different receiving angles at 8GHz.


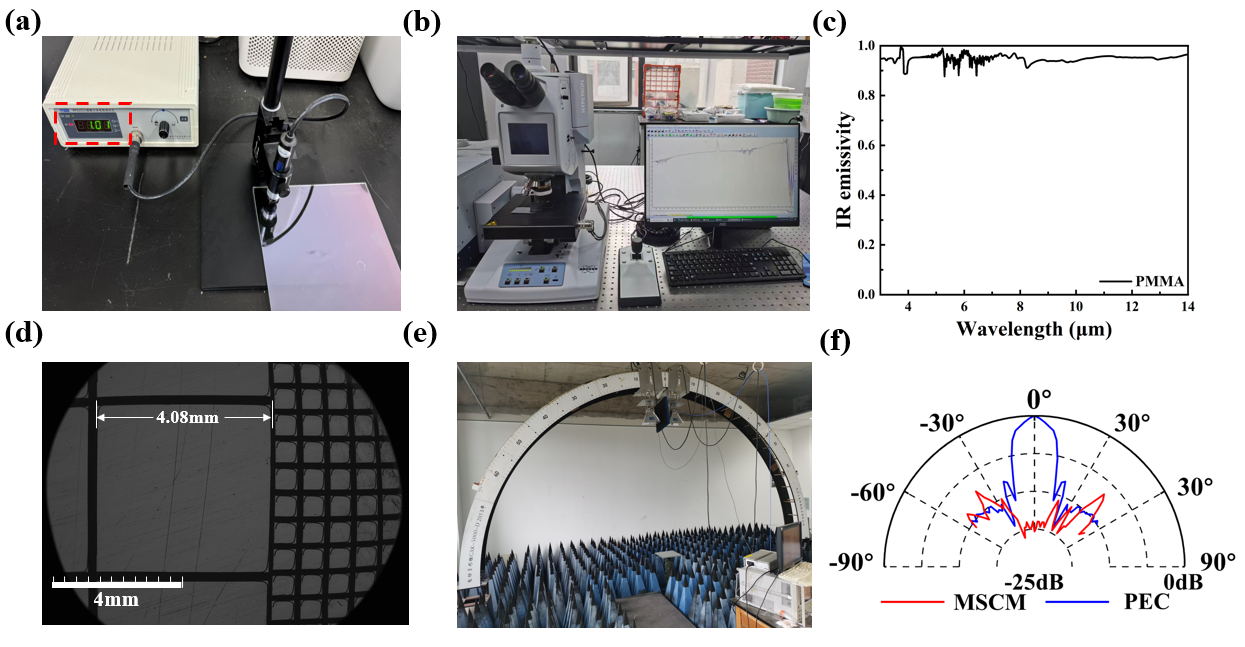


**Figure S17.** (a) Square resistance of IRCM, (b) photo of Hyperion 2000 Fourier transform IR microscope, (c) measured IR emissivity result of PMMA medium, (d) over all processed MSCM under SEM, (e) arch system. (f) measured reflectivity of the MSCM at different receiving angles.

Figure S18 (a) and (b) correspond to the picture when the heating table temperature is 50°C and 90°C, it can be observed that MSCM has a camouflaged IR radiation compared with IRCM and PMMA. Figure S18 (c) and (d) show the picture of MSCM and PMMA at real environment, they both have well visible transparency.


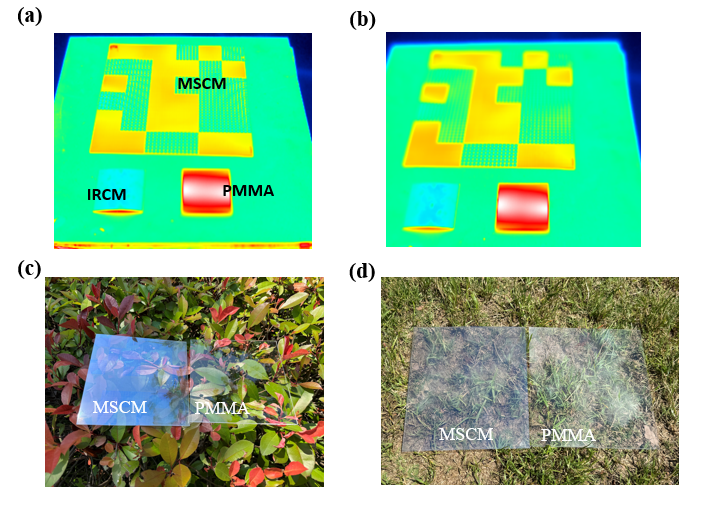


**Figure S18.** Thermal image at (a) 50°C, (b) 90°C. (c), (d) picture of samples on the grass.

**Supporting References:**

[1] Yang H U,D'Archangel J,Sundheimer M L,Tucker E,Boreman G D and Raschke M B 2015 Optical Dielectric Function of Silver. *Physical Review B* ***91*** 235137.

[2] Shkondin E,Takayama O,Panah M E A,Liu P,Larsen P V,Mar M D,Jensen F and Lavrinenko A V 2017 Large-Scale High Aspect Ratio Al-Doped Zno Nanopillars Arrays as Anisotropic Metamaterials. *Opt. Mater. Express* ***7*** 1606-1627.

[3] Johnson P B and Christy R W 1972 Optical Constants of the Noble Metals. *Physical Review B* ***6*** 4370-4379.

[4] Hartnett T M,Bernstein S D,Maguire E A and Tustison R W 1998 Optical Properties of Alon (Aluminum Oxynitride). *Infrared Physics & Technology* ***39*** 203-211.

[5] Born M and Wolf E, *Principles of Optics: 60th Anniversary Edition*, 7 ed.; Cambridge University Press: Cambridge, 2019.

[6] Polyanskiy M N 2024 Refractiveindex.Info Database of Optical Constants. *Scientific Data* ***11*** 94.

[7] Marcuvitz N, *Waveguide Handbook*; McGraw-Hill: New York, 1951.

[8] Fu Y,Ji J,Wang Y,Zhou F,Wang C and Chen P 2022 Broadband Radar Cross Section Reduction Binary Metasurface with a High-Efficiency Intraband Transmission Window. *IEEE Antennas Wirel. Propag. Lett.* ***21*** 878-882.

[9] Fang W,Zhou F,Wang Y and Chen P 2021 Broadband, Wide-Angle, Polarization-Independent and Lightweight Low-Scattering Coding Metamaterial Based on Stereo Meta-Atoms. *Results Phys.* ***20*** 103687.
